# Targeting Nr2e3 to Modulate Tet2 Expression: Therapeutic Potential for Depression Treatment

**DOI:** 10.1002/advs.202400726

**Published:** 2024-06-17

**Authors:** Xiaohua Ma, Shiyao Xu, Yaohui Zhou, Qian Zhang, Hao Yang, Bo Wan, Yong Yang, Zhigang Miao, Xingshun Xu

**Affiliations:** ^1^ Department of Neurology the First Affiliated Hospital of Soochow University Suzhou 215000 China; ^2^ Institute of Neuroscience Soochow University Suzhou 215123 China; ^3^ Department of Fetology the First Affiliated Hospital of Soochow University Suzhou 215006 China; ^4^ Department of Psychiatry the Affiliated Guangji Hospital of Soochow University Suzhou Jiangsu 215000 China; ^5^ Jiangsu Key Laboratory of Neuropsychiatric Diseases Soochow University Suzhou Jiangsu 215123 China

**Keywords:** azacyclonal, depression, Nr2e3, synaptic plasticity, Tet2

## Abstract

Epigenetic mechanisms such as DNA methylation and hydroxymethylation play a significant role in depression. This research has shown that Ten‐eleven translocation 2 (Tet2) deficiency prompts depression‐like behaviors, but Tet2's transcriptional regulation remains unclear. In the study, bioinformatics is used to identify nuclear receptor subfamily 2 group E member 3 (Nr2e3) as a potential Tet2 regulator. Nr2e3 is found to enhance Tet2's transcriptional activity by binding to its promoter region. Nr2e3 knockdown in mouse hippocampus leads to reduced Tet2 expression, depression‐like behaviors, decreased hydroxymethylation of synaptic genes, and downregulation of synaptic proteins like postsynaptic density 95 KDa (PSD95) and N‐methy‐d‐aspartate receptor 1 (NMDAR1). Fewer dendritic spines are also observed. Nr2e3 thus appears to play an antidepressant role under stress. In search of potential treatments, small molecule compounds to increase Nr2e3 expression are screened. Azacyclonal (AZA) is found to enhance the Nr2e3/Tet2 pathway and exhibited antidepressant effects in stressed mice, increasing PSD95 and NMDAR1 expression and dendritic spine density. This study illuminates Tet2's upstream regulatory mechanism, providing a new target for identifying early depression biomarkers and developing treatments.

## Graphical abstract

This study demonstrates that Nr2e3 is a crucial transcription factor of Tet2. Nr2e3 binds to the Tet2 promoter and regulates its transcriptional activity. Nr2e3 knockdown in the hippocampus results in reduced Tet2 expression and depression‐like behaviors in mice. Interestingly, azacyclonol is identified as a compound capable of effectively increasing Nr2e3 protein in the hippocampus and exhibits antidepressant effects in mice. These findings suggest that targeting the Nr2e3/Tet2 pathway holds promise for identifying early depression biomarkers and developing new treatments for depression.

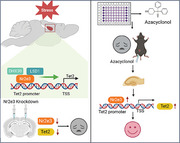

## 1 Introduction

Depression is a severe mental health condition characterized by persistent low mood, loss of interest, and even suicidal tendencies.^[^ ^1^ ^]^ It affects a significant number of people worldwide, with ≈280 million individuals suffering from depression according to the World Health Organization.^[^ ^2^ ^]^ However, currently available pharmacotherapies for depression usually take several weeks to show effects and only benefit ≈60% of patients.^[^ ^3^ ^]^ There is an urgent need for antidepressants that have faster onset and broader efficacy across different types of depression. Therefore, understanding the pathogenesis of depression is crucial for the development of effective antidepressant drugs and remains a major focus of research in the field.

Recent studies have highlighted the significant role of epigenetic mechanisms in the pathogenesis of depression.^[^ ^4^ ^]^ Epigenetic modifications are the production of crosstalk of environmental stimuli and susceptibility genes in response to stress. Specifically, DNA methylation (5mC) and hydroxymethylation (5hmC) have been implicated in the development of depression.^[^ ^5^ ^]^ These epigenetic modifications contribute to depression by regulating the transcriptional activity of genes associated with the condition, such as brain‐derived neurotrophic factor (BDNF) and Glutathione Reductase (GR).^[^ ^6^, ^7^ ^]^ The ten‐eleven translocation (Tet) family of enzymes, including Tet1, Tet2, and Tet3play a crucial role in depression by facilitating the conversion of 5mC to 5hmC in DNA and RNA. For example, Tet1 deficiency enhances the resistance to chronic restraint stress, while Tet2 deficiency increases the susceptibility to chronic restraint stress.^[^ ^8^ ^]^ Loss of Tet3 in the nucleus accumbent amplifies anxiety‐like behaviors.^[^ ^9^ ^]^ Among these enzymes, Tet2 is considered to be the most closely related to the occurrence of depression because knockout (KO) of Tet2 leads to significant depression‐like behaviors,^[^ ^7^, ^10^, ^11^ ^]^ and affects the occurrence of post‐stroke depression by regulating the Wnt/β‐Catenin/LEF1 pathway.^[^ ^12^ ^]^ Recent studies have also demonstrated that Tet2 is involved in depressive behaviors by regulating the 5hmC modification of downstream genes, such as erythropoietin, lymphoid enhancer factor 1, and BDNF.^[^ ^8^, ^13^ ^]^ In addition, Tet2 in the lateral habenula modulates the expression of the Sh3rf2 protein by altering the 5hmC modification of the Sh3rf2 gene, thereby influencing social preference.^[^ ^14^ ^]^ Given the importance of Tet2 expression in maintaining normal brain function, understanding the transcriptional regulation of Tet2 in depression is of great interest.

In our previous study, we found that Tet2 expression is highly increased in the hippocampus after environmental stress.^[^ ^11^ ^]^ Due to depressive behaviors in Tet2 KO mice, the adaptive increase of Tet2 expression in the brain upon stress stimuli may be a protective mechanism for the maintenance of normal behaviors. Targeting Tet2 expression in the brain could be a promising therapeutic strategy for abnormal behaviors, including depression and social defects. Therefore, understanding the transcriptional regulation of Tet2, specifically the identification of its transcription factor, is crucial for advancing translational research in this area. In this study, we have identified the transcription factor Nr2e3 as the key mediator for Tet2 expression in the brain. By up‐regulating Nr2e3 levels through the use of small molecules, we observed an increase in Tet2 expression and a recovery of depressive behaviors in mice. These findings support our hypothesis that targeting the Nr2e3/Tet2 pathway could be a viable therapeutic strategy for addressing abnormal behaviors, such as depression and social deficits.

## 2 Results

### 2.1 Nr2e3 was a Potential Transcription Factor of Tet2

**Figure 1:**
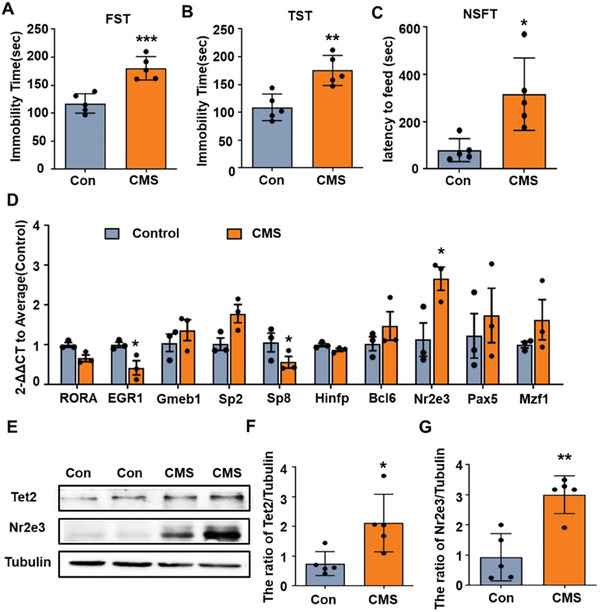
Nr2e3 was selected as a potential transcriptional factor of Tet2 expression. A–C) Mice were treated with different stressors daily to induce CMS mice. The FST (A), the TST (B) and the NSFT (C) were performed on control mice and CMS mice (*n* = 5). D) The hippocampal tissues were collected from control mice and CMS mice. The mRNA levels of 10 potential transcription factors of Tet2 were examined by qPCR analysis on control and CMS mice (*n* = 5). E) Tet2 and Nr2e3 protein levels were examined by Western blot. Representative blots of Tet2 and Nr2e3 protein after CMS were shown. F, G) Statistical analysis of F) Tet2 protein level and G) Nr2e3 level were shown (*n* = 5). All data were presented as mean ± SEM. A, B, C, F, G by using Student's *t*‐test. D)by using two‐way ANOVA with Tukey's multiple comparison tests (*p < 0.05, **p < 0.01, and ***p < 0.001).

To explore the potential transcription factors that bind to the Tet2 promoter and regulate the gene expression of Tet2, we analyzed the Tet2 promoter sequences to search for transcription factors by using the bioinformatic software including Jaspar, animal TFDB, and hTF target. We identified 119 candidate genes and further analyzed these genes by using Gene Ontology (GO) enrichment analyses (Figure S1A, Supporting Information). The results showed that these genes were involved in many biological processes as shown in Figure S1B (Supporting Information). We focused on biological processes related to transcriptional regulation in the central nervous system and stress, including DNA transcription, sensory organ development, brain‐derived neurotrophic factor signaling pathway, and cellular response to hormone stimulus (Figure S1B, Supporting Information). Combined with the score of the transcription factor binding motif, ten transcription factors were selected for further study. We examined the gene expression of ten genes in the hippocampus of mice with chronic mild stress (CMS). Compared with control mice, the CMS mice exhibit significantly increased immobility time in forced swimming test (FST) and tail suspension test (TST), and spend more time in novelty‐suppressed feeding test (NSFT) (**Figure** **1**A–C). The quantitative PCR (qPCR) results showed that the expressions of three transcription factors were markedly altered in CMS mice. Among these genes, early growth response 1 (Egr1) and specificity protein 8 (Sp8) were decreased, while Nr2e3 was significantly increased (Figure 1D). Based on these findings, we hypothesized that Nr2e3 may be the primary regulator of Tet2 in the context of depression. However, additional research is required to ascertain whether Egr1 and Sp8 also have a regulatory role in Tet2 expression under stress conditions. Therefore, we further demonstrated whether Nr2e3 increased Tet2 expression during CMS. We confirmed that the protein levels of Nr2e3 and Tet2 were increased in the hippocampus of CMS mice (Figure 1E–G). In addition, we found that Nr2e3 was more abundant in the brainstem (BS), the hippocampus (HIP), and the cortex (CTX) than in other regions like the hypothalamus (HYP), the prefrontal cortex (PFC), the cerebellum (CERE), the striatum (STR), the olfactory bulb (OB), and the amygdala (AMY) (Figure S2A,B, Supporting Information). In addition, we also observed significantly higher expression of Nr2e3 protein in the retina compared to the hippocampus (Figure S2A,B, Supporting Information). Therefore, we investigated the expression of Nr2e3 in other depression‐related brain regions except the hippocampus. However, no significant change was observed in the HYP, the PFC, and the CTX after CMS stress (Figure S2C–E, Supporting Information). Therefore, we chose the hippocampus for further research.

### 2.2 Nr2e3 Regulated the Gene Expression of Tet2

**Figure 2:**
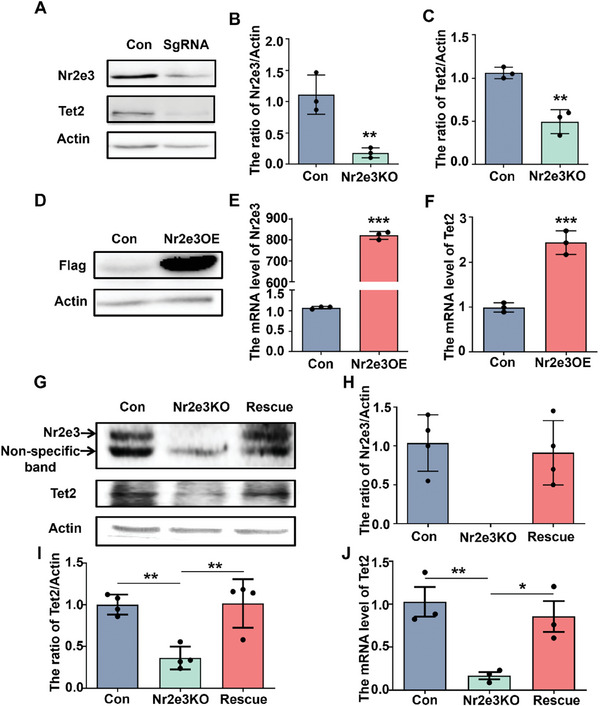
Nr2e3 positively regulated Tet2 expression in HT‐22 cells. A) After knockdown Nr2e3 in HT22 cells through transient infection of sgRNA and Cas9 expression lentivirus, Tet2 and Nr2e3 protein levels were examined by Western blot. Representative blots of Tet2 and Nr2e3 proteins were shown. B, C) Quantitative analysis of B) Nr2e3 protein level and C) Tet2 protein level were performed (*n* = 3). D) HT22 cells were infected with Nr2e3‐3×Flag lentivirus. After puromycin screening and single clone isolation, the Nr2e3 protein level in Nr2e3OE cells was determined by Western blot with an anti‐flag antibody. E) Nr2e3 and F) Tet2 mRNA levels in normal HT22 cells (Con) and Nr2e3OE cells were analyzed by qPCR (*n* = 3). G) The protein levels of Nr2e3 and Tet2 were examined by Western blot in normal HT22 cells (Con), HT22 Nr2e3KO cells, and HT22 Nr2e3KO cells infected with Nr2e3 lentivirus (rescue cells). H) Nr2e3 protein levels in different cells was analyzed (*n* = 4). I) Quantitative analysis of Tet2 protein level was performed (*n* = 4). J) qPCR analysis of Tet2 mRNA level in different cells were examined (*n* = 3). All data were presented as mean ± SEM. B, C, E, F) by using Student's *t*‐test. H, I, J) by using two‐way ANOVA with Tukey's multiple comparison tests (*p < 0.05, **p < 0.01 and ***p < 0.001).

We further explored whether Nr2e3 regulates the expression of Tet2. First, we knocked down the expression of Nr2e3 by infecting HT22 cells with sgRNA and Cas9 expression lentivirus. The results showed that Nr2e3 was knocked down in HT22 cells (**Figure** **2**A,B). At the same time, the protein level of Tet2 was also significantly decreased in HT22 cells (Figure 2A,C). Similarly, siRNA targeting Nr2e3 decreased the Nr2e3 mRNA in Neuron‐2a cells, and the reduction of Tet2 mRNA was also observed (Figure S3A,B, Supporting Information). On the contrary, Nr2e3 overexpression (OE) in HT22 cells significantly increased the protein and mRNA levels of Nr2e3 (*p *< 0.05, Figure 2D,E). Tet2 mRNA (*p *< 0.05, Figure 2F) and Tet2 protein (*p *< 0.05, Figure S3C,D, Supporting Information) were also markedly increased in cells. These results indicated that the expression of Tet2 was regulated by Nr2e3. To further confirm this hypothesis, we isolated a single clone of the Nr2e3KO HT22 cell line, in which 102 bp of Nr2e3 genomic DNA was deleted, 88 bp of exon 3 and 14 bp of intron 3 (Figure S4A,B, Supporting Information). To rescue the expression of Nr2e3, Nr2e3KO cells were further infected with Nr2e3 lentivirus (rescued cells), and then the expression of Nr2e3 and Tet2 was analyzed. Western blot results showed that Nr2e3 protein was not detected in Nr2e3KO cells, but Nr2e3 lentivirus infection increased Nr2e3 protein levels in rescued cells (Figure 2G,H). Tet2 protein also significantly decreased in Nr2e3KO cells but increased in rescued cells after the infection with Nr2e3 lentivirus (Figure 2G,I). Moreover, the Tet2 mRNA level also significantly decreased in Nr2e3KO cells and increased in rescued cells (Figure 2J). As a catalytic product of the Tet enzyme, 5hmC is often used to evaluate the activity of the Tet enzyme. We also examined the levels of 5hmC in HT22 cells, Nr2e3KO cells, and rescue cells by dot blot analysis. As shown in Figure S5A,B (Supporting Information), the levels of 5hmC were reduced in Nr2e3KO cells compared to HT22 control cells. In contrast, the levels of 5hmC were increased in rescue cells compared to Nr2e3KO cells. To rule out the possibility that Nr2e3 expression is regulated by Tet2, we detected the protein and mRNA levels of Nr2e3 in the hippocampus of Tet2 cKO mice. The results showed that the protein and mRNA levels of Nr2e3 were not changed in Tet2 cKO mice compared with WT mice, indicating that Nr2e3 was not regulated by Tet2 (Figure S6A–C, Supporting Information).

### 2.3 Nr2e3 Directly Bound to the Tet2 Promoter and Enhanced the Transcriptional Activity of Tet2 Promoter

**Figure 3:**
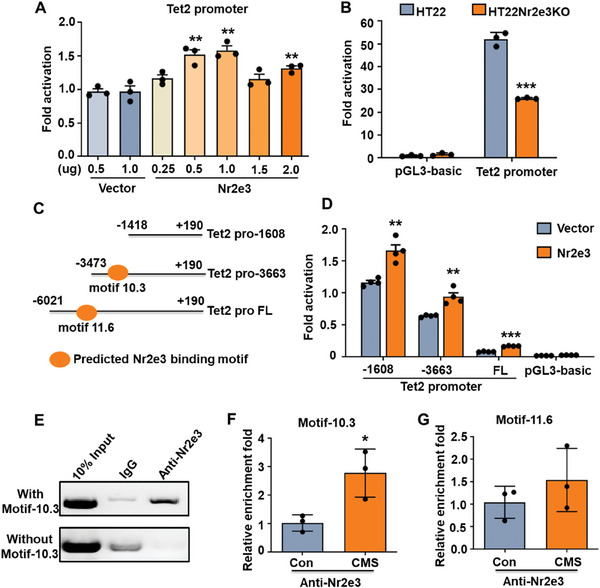
Nr2e3 bound to the Tet2 promoter directly and enhanced the activity of the Tet2 promoter. A) Different doses (0.25, 0.5, 1.0, 1.5, and 2.0 µg) of Nr2e3 plasmid were co‐transfected with full‐length Tet2 promoter (Tet2 pro FL) in 293T cells. Dual‐luciferase reporter assay was performed to measure the activity of the Tet2 promoter at 48 h after transfection (*n* = 3). B) Full‐length Tet2 promoter plasmid was transfected into HT22 Nr2e3KO cell line. Dual‐luciferase reporter assay was performed at 48 h after transfection (*n* = 3). C) The predicted Nr2e3 binding motifs (motif 10.3 and motif 11.6) at the Tet2 promoter by Jaspar (vs UCSD NCBI37 mm9) were shown. Different Tet2 promoter plasmids containing different truncated Tet2 promoters were constructed. D) Truncated Tet2 promoter plasmid and control plasmids were transfected into 293T cells. Dual‐luciferase reporter assay was performed to measure the activity of Tet2 promoter at 48 h post‐transfection (*n* = 3). E) Full‐length Tet2 promoter plasmid (with motif‐10.3) and Tet2 promoter plasmid without motif‐10.3 (Tet2 pro‐1608) were separately co‐transfected into HEK293T cells with Nr2e3 plasmid. Nr2e3 monoclonal antibody and IgG antibody were used to perform the CHIP. The captured genomic DNA by Nr2e3 antibody was used to amplify PCR product containing Tet2 motif‐10.3. F, G) The hippocampus of CMS mice and control mice were collected. Nr2e3 monoclonal antibody was used to perform the CHIP‐qPCR in the hippocampal lysates. The enrichment fold changes of F) motif‐10.3 and G) motif‐11.6 were analyzed by qPCR (*n* = 3). All data were presented as mean ± SEM. A) by using one‐way ANOVA with Tukey's multiple comparison tests. B, D) by using two‐way ANOVA with Tukey's multiple comparison test. F, G by using Student's *t*‐test (*p < 0.05, **p < 0.01, and ***p < 0.001).

To explore the mechanism by which Nr2e3 enhances the expression of Tet2, we investigated the effect of Nr2e3 on the transcriptional activity of the Tet2 promoter by co‐transfecting Nr2e3 plasmid and full‐length Tet2 promoter plasmid. Our dual‐luciferase reporter assay results indicated that Nr2e3 enhanced the transcriptional activity of the Tet2 promoter (**Figure** **3**A). However, when full‐length Tet2 promoter plasmid was transfected into HT22 Nr2e3KO cells, the transcriptional activity of the Tet2 promoter was downregulated in the HT22 Nr2e3KO cell line (*p *< 0.05, Figure 3B). Previous studies have shown that Nr2e3 directly binds to the promoter region of genes through recognizing nucleotide sequence motifs.^[^ ^15^ ^]^ Therefore, we determined whether Nr2e3 directly binds to the Tet2 promoter by recognizing nucleotide sequence motifs. The potential Nr2e3 binding motifs of the Tet2 promoter were predicted by the Jaspar software in the −6021 to +190 of Tet2 transcription start site (TSS); two highest scores of binding motifs were found: motif‐10.3 (AAAGCTT, base position −3226 to −3233) and motif‐11.6 (AAGCTTG base position −5668 to −5675). The different plasmids carrying truncated Tet2 promoters to contain different putative Nr2e3 binding motifs were constructed as shown in Figure 3C. The results of the dual‐luciferase reporter assay showed that Nr2e3 increased the activity of all three Tet2 promoter constructions, including the Tet2 pro‐1608 which lacked the motif 10.3 (Figure 3D). To investigate the major binding motif of Nr2e3 to Tet2 promoter, the enrichment of two motifs was detected through chromatin immunoprecipitation PCR (CHIP‐PCR). HEK293T cells were transfected with the Tet2 promoter reporter plasmid and Nr2e3 plasmid, and then CHIP‐PCR was performed using Nr2e3 monoclonal antibody. The result showed that the target region containing motif‐10.3 was significantly enriched by the anti‐Nr2e3 antibody (Figure 3E upper panel). In contrast, when Tet2 promoter plasmid (Tet2 pro‐1608) without motif‐10.3 and Nr2e3 plasmid were transfected in HEK293T cells, Nr2e3‐CHIP data showed that the target band was clearly observed in the input, but not in the Nr2e3 antibody‐treated samples (Figure 3E lower panel). These results suggested that motif‐10.3 may be the major binding site of Nr2e3 to the Tet2 promoter. Considering Tet2 and Nr2e3 were both upregulated in the hippocampus of CMS mice, we investigated whether the binding of Nr2e3 to the motif‐10.3 was also increased in stressed mice. CHIP‐quantitative PCR (CHIP‐qPCR) results showed that the enrichment of motif‐10.3, but not motif‐11.6, was significantly increased in CMS mice compared with control mice (Figure 3F,G). Therefore, we identified motif‐10.3 of the Tet2 promoter region as the major binding site of Nr2e3 to enhance the transcriptional activity of Tet2.

### 2.4 DHX30 and LSD1 Cooperated with Nr2e3 to Regulate Tet2

**Figure 4:**
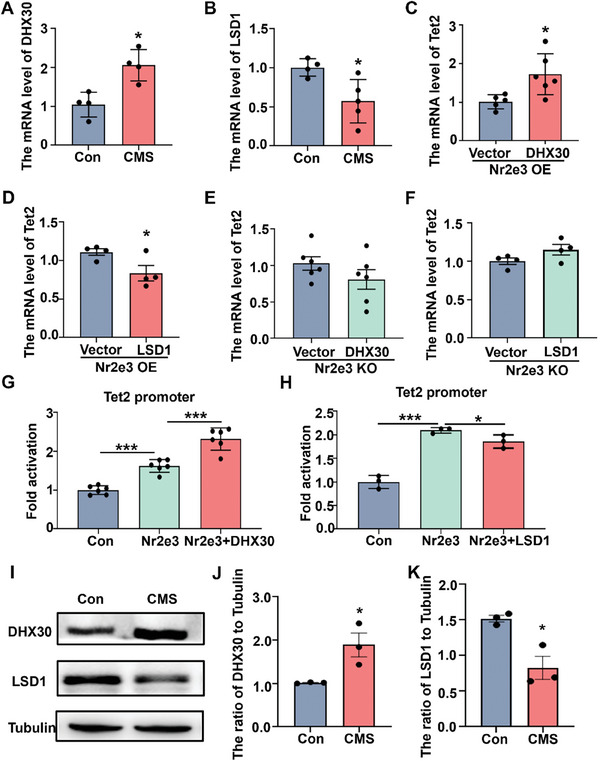
Nr2e3 co‐regulated the expression of Tet2 with DHX30 and LSD1. A, B) The hippocampal tissues of control mice and CMS mice were collected, then protein and mRNA were extracted. DHX30 mRNA level (A) and LSD1 mRNA level (B) were examined by qPCR analysis (*n* = 4). C) DHX30 plasmid was transfected in HT22 Nr2e3OE cells, and Tet2 mRNA level was examined by qPCR analysis at 48 h after transfection (*n* = 5–6). D) LSD1 plasmid was transfected in HT22 Nr2e3OE cells. Tet2 mRNA level was examined by qPCR analysis at 48 h after transfection (*n* = 4). E) DHX30 plasmid was transfected in HT22 Nr2e3KO cells, and then the Tet2 mRNA level was examined by qPCR analysis at 48 h after transfection (*n* = 6). F) LSD1 plasmid was transfected in HT22 Nr2e3KO cells. Tet2 mRNA level was examined by qPCR analysis at 48 h after transfection (*n* = 4). G) DHX30 plasmid was co‐transfected with Nr2e3 in 293T cells. Tet2 promoter activity was detected using dual‐luciferase reporter assay at 48 h after transfection (*n* = 3). H) LSD1 plasmid was co‐transfected with Nr2e3 in 293T cells. Dual‐luciferase reporter assay was performed to detect Tet2 promoter activity at 48 h after transfection (*n* = 3). I) DHX30 and LSD1 protein levels in the hippocampus were examined by Western blot following CMS. J, K) quantitative analysis of J) DHX30 and K) LSD1 protein levels was performed in control mice and CMS mice (*n* = 3). All data were presented as mean ± SEM. A–F, J, K) by using Student's *t*‐test. G, H) by using two‐way ANOVA with Tukey's multiple comparison tests (*p < 0.05 and ***p < 0.001).

Previous studies have shown that Nr2e3 needs some co‐regulators including neural retina leucine zipper (Nrl), SIN3 transcription regulator family member A (Sin3A), lysine‐specific histone demethylase 1 (LSD1), nuclear receptor subfamily 1 group D member 1 (NR1D1), cone‐rod homeobox (CRX), nuclear receptor coactivators (NCOA1, NCOA2, NCOA3) and DEAH box polypeptide 30 (DHX30) to function together in rod cells or tumor cells.^[^ ^16^, ^17^, ^18^, ^19^, ^20^ ^]^ To explore whether these co‐regulators also contribute to the regulation of Tet2, we first detected the mRNA levels of these nine co‐regulators in the hippocampal tissues of CMS mice. The mRNA levels of DHX30, NrL, NR1D1, and CRX were significantly increased (**Figure** **4**A; Figure S7, Supporting Information), and LSD1 was decreased (Figure 4B). We hypothesized that these five co‐regulators may cooperate with Nr2e3 to regulate Tet2 under stress. To confirm this hypothesis, we detected the mRNA levels of Tet2 in Nr2e3OE cells and Nr2e3KO cells after transfection with co‐factor plasmids. In the Nr2e3OE cells, DHX30, NR1D1, NCOA1, and NCOA3 significantly increased the mRNA levels of Tet2 (Figure 4C; Figure S8A, Supporting Information). On the contrary, LSD1 markedly decreased the mRNA level of Tet2 (Figure 4D). However, all cofactors failed to affect the expression of Tet2 in the Nr2e3KO cells (Figure 4E,F; Figure S8B, Supporting Information). To further examine the effect of these factors on the transcriptional activity of the Tet2 promoter, we performed the dual‐luciferase reporter assay by transfecting co‐factor plasmids and Nr2e3 plasmid as well as the Tet2 promoter plasmid. We found that compared with Nr2e3 transfection alone, only DHX30 significantly further increased the transcriptional activity of the Tet2 promoter (Figure 4G), while LSD1 decreased the transcriptional activity of the Tet2 promoter (Figure 4H; Figure S9, Supporting Information), indicating that DHX30 and LSD1 participated the regulation on Tet2 expression along with Nr2e3. It is worth noting that the mRNA level of DHX30 increased, and that of LSD1 decreased after CMS stress (Figure 4A,B). Additionally, the protein level of DHX30 also increased, and that of LSD1 decreased following CMS stress (Figure 4I,K). This may explain the reason why Tet2 was increased after stress as described previously.^[^ ^11^ ^]^

### 2.5 Knockdown of Nr2e3 in the Hippocampus Caused Depression‐Like Behaviors in Mice

**Figure 5:**
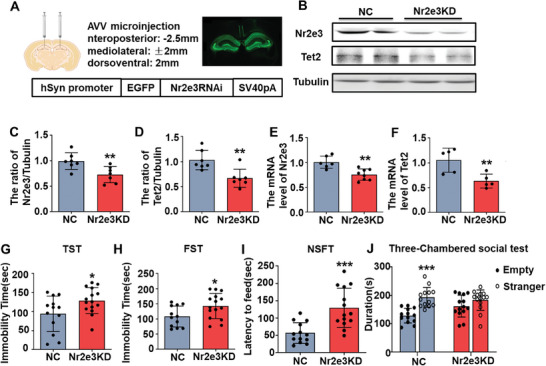
The knockdown of Nr2e3 in the hippocampus resulted in depression‐like behaviors in mice. A) A schematic diagram showed virus injection sites and the structure of the AAV plasmid. B) After 28 d of AAV‐Nr2e3 RNAi virus injection into both sides of the hippocampus, protein levels of Nr2e3 and Tet2 were examined by Western blot. C, D) Quantitative analysis of C) Nr2e3 and D) Tet2 protein levels was performed (*n* = 7). E, F) At the same time, E) Nr2e3 and F) Tet2 mRNA levels were examined by qPCR at 28 d after viral injection (*n* = 5). G–I) The TST (G), the FST (H) and the NSFT (I) were performed from 21 d after viral injection (*n* = 15). L) The three‐chamber social behavioral tests were also examined at 24 d after viral injection (*n* = 15). All data were presented as mean ± SEM. C–I) by using Student's *t*‐test. J by using two‐way ANOVA with Tukey's multiple comparison tests (*p < 0.05, **p < 0.01, and ***p < 0.001).

Because deficiency of Tet2 causes depressive behaviors in mice, we further determined whether knockdown Nr2e3 in the hippocampus also causes depression‐like behaviors in mice. Nr2e3 was knocked down in the bilateral hippocampus by injecting the AAV virus that contained hSyn promoter and Nr2e3‐shRNA (**Figure** **5**A). At 3 weeks after injection of AAV virus, the protein and mRNA levels of Nr2e3 were decreased in the hippocampus of Nr2e3 shRNA virus‐treated mice (Nr2e3KD) (Figure 5B,C,E). Accordingly, the protein and mRNA levels of Tet2 were also decreased after the virus injection (Figure 5B,D,F). Furthermore, the 5hmC levels significantly decreased after Nr2e3 knockdown in the hippocampal tissues (Figure S5C,D, Supporting Information). Behavior tests were carried out at 3 weeks post viral injection. Nr2e3KD mice showed longer immobility time in TST and FST (Figure 5G,H), and spent more time finding food in NSFT (Figure 5I). The results of three‐chamber social tests showed that the social time between Nr2e3KD mice and stranger mouse 1 was reduced, indicating that Nr2e3KD mice had social preference defects (Figure 5J). However, in the open field test (OFT), Nr2e3KD mice did not show anxiety‐like behaviors (Figure S10A–E, Supporting Information). In addition, we injected Nr2e3 RNAi AAV or control AAV into the bilateral hippocampus of Tet2 cKO mice. TST and FST were tested at days 7 and 14 after virus injection according to literature reports that AAV9 exhibits high expression levels at 7 d post‐injection.^[^ ^21^ ^]^ TST and FST results showed that depressive behaviors were not changed in Nr2e3KD mice compared with control AAV‐treated mice (Figure S11A,B, Supporting Information). These results support that Nr2e3 is involved in the mechanism of depressive behaviors by regulating the expression of Tet2.

### 2.6 Knockdown of Nr2e3 Altered the Hippocampal DNA 5hmC Landscape

**Figure 6:**
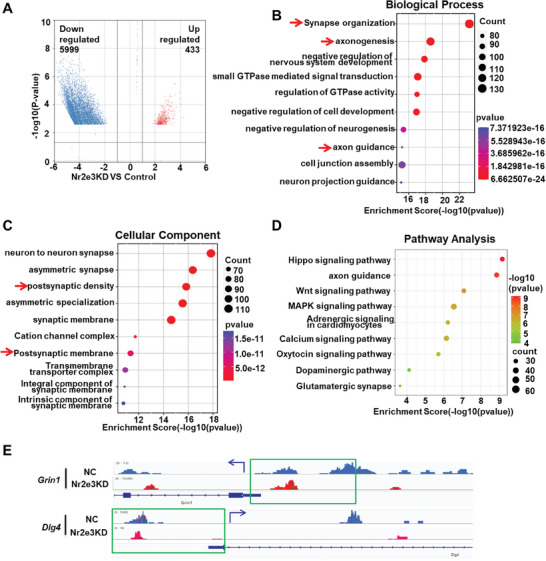
The 5hmC modification changes in genes were analyzed in AAV‐Nr2e3 RNAi‐treated mice. A) The hippocampal tissues from AAV‐Nr2e3 RNAi‐treated (Nr2e3 KD) mice and control AAV‐RNAi‐treated (negative control, NC) mice were harvested and genomic DNA was extracted for whole genome 5hmC sequencing. Volcano plot analysis of genome‐wide 5hmC‐sequencing data was performed(N = 3). The blue dots represented the 5hmC downregulated DhMLs, while the red dots represented the 5hmC upregulated DhMLs. B, C) For these downregulated DhMLs, GO enrichment analyses were performed. The B) top 10 biological processes and C) top 10 cell components were shown respectively. D) Kyoto Encyclopedia of Genes and Genomes (KEGG) analysis was performed in 5hmC downregulated genes. The top 10 signal pathways were displayed. E) 5hmC dynamic changes in the promoter region (−2000 – +200 of TSS) of Grin1 (NMDAR1) and Dgl4 (PSD95) in NC mice and Nr2e3 KD mice were visualized by an integrated genomics viewer (IGV). The blue arrows indicated the transcription direction of the gene, and the green boxes indicated the promoter region.

We further analyzed the differentially hydroxymethylated loci (DhMLs) in the hippocampus of Nr2e3KD mice by genome‐wide analysis of DNA 5hmC modifications. We first analyzed the DhMLs between the control and Nr2e3KD mice. Compared with control mice, 5999 DhMLs had decreased 5hmC modification and were mapped to 5243 genes in Nr2e3KD mice, while only 433 DhMLs gained 5hmC modification and were mapped to 43 genes (**Figure** **6**A). The GO analysis result of 5243 genes showed that these genes were mainly enriched in several synapse‐associated biological processes, such as synapse organization, axonogenesis, and axon guidance (Figure 6B). Cellular component analysis suggested that 5243 genes were mainly located in the synaptic membrane and postsynaptic density (Figure 6C). Moreover, the KEGG pathway analysis revealed that these genes were significantly enriched in pathways in the hippo signal pathway, axon guidance, dopaminergic synapse, and glutamatergic synapse (Figure 6D). These results indicated that Nr2e3 may be involved in synaptic plasticity through the regulation of Tet2. Considering that the 5hmC modification in the promoter region may more effectively contribute to transcription, we analyzed the 5hmC distribution in the promoter region of *Grin1* and *Dlg4* because *Grin1* encodes NMDAR1 protein and *Dlg4* encodes PSD95 protein, two proteins are both tightly related to synaptic plasticity. The results in Integrative Genomics Viewer showed that 5hmC modification distributed in the promoter of both genes was reduced (Figure 6E).

### 2.7 Knockdown of Nr2e3 Reduced the Dendritic Spines of Hippocampal Neurons

**Figure 7:**
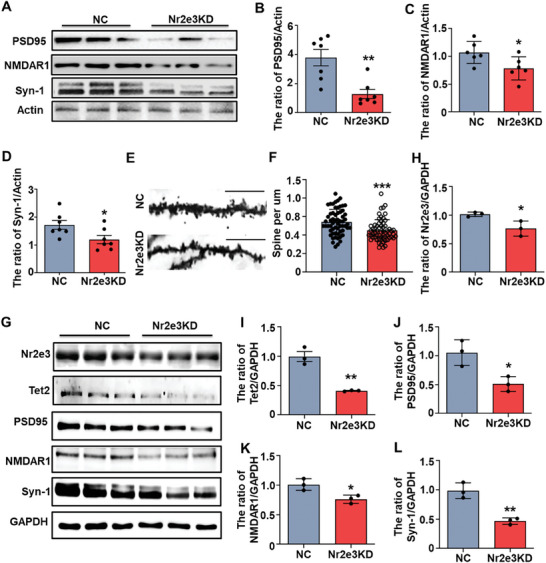
Nr2e3‐Tet2 signal pathway was involved in hippocampal neuronal plasticity. A) The hippocampal tissues from Nr2e3 KD mice and control (NC) mice were collected for Western blot. Protein levels of PSD95, Syn1, and NMDAR1 in the hippocampus were detected. B–D) Protein levels of PSD95 (B, *n* = 7), NMDAR1 (C, *n* = 6), and Syn1 (D, *n* = 7) in the hippocampus were quantitatively analyzed. E) The spines of hippocampus neurons in control mice and Nr2e3 KD mice by Golgi staining were shown (scale bar, 20um). F) The quantitative analysis of spine number in the hippocampal neurons was performed (*n* = 60). G) Primary neurons were isolated and cultured for 4 d, and then the neurons were infected with AAV‐Nr2e3 RNAi and control AAV‐RNAi for 12 h. After an additional 8 d of culture, the neurons were harvested. The protein levels of Nr2e3, Tet2, PSD95, NMDAR1, and Syn1 were detected by Western blot. H–L) Quantitative analysis of protein levels of H) Nr2e3, I) Tet2, J) PSD95, K) NMDAR1, L) and Syn1 was performed (*n* = 3). All data were presented as mean ± SEM. Statistical significance was determined by using Student's *t*‐test (*p < 0.05, **p < 0.01, and ***p < 0.001).

Given the reduction of the 5hmC modification in the promoter region of *Grin1* and *Dlg4*, we detected the protein expression of NMDAR1 and PSD95 and found that both NMDAR1 and PSD95 proteins were decreased in the hippocampus of Nr2e3KD mice (**Figure** **7**A–C). We also found that synapsin‐1 (Syn‐1) was also decreased in the hippocampus of Nr2e3KD mice (Figure 7A,D). The dendritic spine density of the hippocampus of mice was also detected after Nr2e3 knockdown by using Golgi staining. The results showed that the density of dendritic spines decreased markedly in the hippocampus of Nr2e3KD mice (Figure 7E,F). Similarly, when Nr2e3 was knocked down by AAV‐mediated RNA interference in primary neurons, Tet2 was also decreased (Figure 7G–I). PSD95, NMDAR1, and Syn‐1 were all decreased in Nr2e3KD neurons (Figure 7G,J–L).

### 2.8 AZA Increased Nr2e3 Expression in the Brain and Improved Depression‐Like Behaviors in Mice

**Figure 8:**
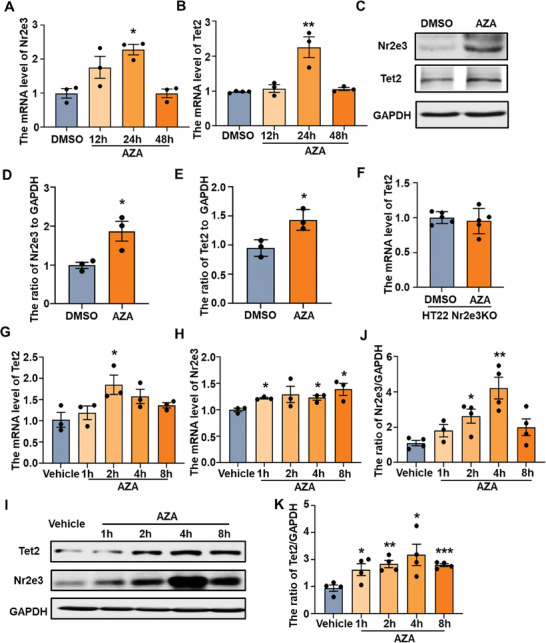
AZA increased Nr2e3 expression in HT22 cells and mice. A, B) HT22 cells were treated with AZA (10 µm) or DMSO. At different time points after AZA treatment (12, 24, and 48 h), cells were harvested, and A) Nr2e3 and B) Tet2 mRNA levels were examined by qPCR analysis. C. HT22 cells were treated with AZA (10 µm) or DMSO for 24 h. Protein levels for Tet2 and Nr2e3 were examined by Western blot. D, E) Quantitative analysis of protein levels for D) Nr2e3 and E) Tet2 was performed (*n* = 3). F) HT22 Nr2e3KO cells were treated with AZA (10 µm) or DMSO for 24 h. The mRNA level of Tet2 was examined by qPCR analysis. G, H) Mice were treated with AZA (5 mg kg^−1^ i.p) or same volume of vehicle. At different time points (1 h, 2 h, 4 h and 8 h) after AZA administration, the hippocampal tissues were collected. G) Tet2 and H) Nr2e3 mRNA levels was examined by qqPCR analysis (*n* = 3). I) At the same time, protein levels for Tet2 and Nr2e3 at different time points (1, 2, 4, and 8 h) after AZA treatment were examined by Western blot analysis. J, K) Protein levels for J) Nr2e3 and K) Tet2 were quantified (*n* = 4). All data were presented as mean ± SEM. A, B, G, H, J, K by using one‐way ANOVA with Tukey's multiple comparison tests. D, E, F by using Student's *t*‐test (*p < 0.05, **p < 0.01, and ***p < 0.001).

**Figure 9:**
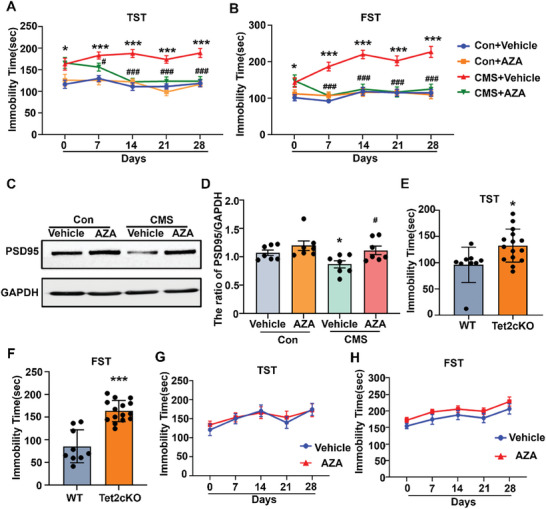
AZA improved depression‐like behaviors via the Nr2e3‐Tet2 signaling pathway. A, B) Control mice and CMS mice were treated with AZA (5 mg kg^−1^ i.p) or the same volume of vehicle for 4 w. TST (A) and FST (B) were performed weekly (*n* = 10). C) After AZA administration for 28 d‐, the hippocampal tissues were collected. Protein levels of PSD95 were determined by Western blot. D) Protein levels of PSD95 were quantified (*n* = 7). E) TST and F) FST were performed in control mice and Tet2 cKO mice (*n* = 9,15). G, H) Tet2 cKO mice were treated with AZA (5 mg kg^−1^ i.p) or the same volume of vehicle. G) TST and H) FST were performed weekly (*n* = 7–8). All data were presented as mean ± SEM. A, B, D, G, H) by using two‐way ANOVA with Tukey's multiple comparison tests. E, F) by using Student's *t*‐test (*p < 0.05, **p < 0.01 and ***p < 0.001 vs Con+vehicle group or WT group; ^#^ *p* < 0.05 and ^###^ *p *< 0.001 vs CMS+ vehicle group).

Since Nr2e3 may play an antidepressant role in depression, we aimed to identify Nr2e3 as a therapeutic target of antidepressant drug development. Therefore, we screened small molecules to increase cellular Nr2e3 level by a cell‐based method. We utilized a compound bank provided by Selleck Chemicals Company to screen molecules aimed at increasing Nr2e3 expression in HT22 cells by incubation and subsequently examining Nr2e3 protein levels through Western blot analysis. Among these compounds, seven compounds were found to increase the mRNA level of Nr2e3, and four of them were accompanied by the increase of Tet2 mRNA (Figure S12A,B, Supporting Information). One of the compounds, azacyclonol (AZA, S3196), was selected for further research. AZA, an isomer of piperonyl alcohol, also known as γ‐Pipebenzyl alcohol, has been previously associated with reducing hallucinations caused by hallucinogens LSD and mescaline, inhibiting mental arousal caused by benzyl alcohol, and demonstrating minimal side effects.^[^ ^22^, ^23^ ^]^ When HT22 cells were treated with AZA, Nr2e3 and Tet2 were increased significantly at 24 h and returned to normal levels at 48 h (**Figure** **8**A,B). Similarly, protein levels of Nr2e3 and Tet2 were also increased significantly at 24 h after AZA treatment (Figure 8C–E). However, AZA did not increase the expression of Tet2 in Nr2e3KO HT22 cells (Figure 8F), suggesting that AZA promoted the expression of Tet2 in an Nr2e3‐dependent manner. We further determined whether AZA enhanced the expression of Nr2e3 and Tet2 in vivo. The mRNA level of Tet2 in the hippocampus was significantly increased at 2 h after AZA injection (5 mg kg^−1^ i.p) (Figure 8G), however, the mRNA level of Nr2e3 in the hippocampus was maintained at a high level at several time points (Figure 8H). The protein levels of Nr2e3 and Tet2 in the hippocampus were also maintained at a high level at several time points after AZA injection (Figure 8I–K). Subsequently, to investigate whether AZA had an antidepressant effect, stressed mice were injected with AZA (5 mg kg^−1^ i.p daily) for 4 weeks, and behavioral tests were performed each week. After 1‐week treatment, the immobility times of AZA‐treated stressed mice in TST and FST significantly reduced compared with vehicle‐treated stressed mice (**Figure** **9**A,B). In addition, the expression of PSD95 was decreased in mice after CMS and was reversed by AZA treatment (Figure 9C,D). To investigate whether the Nr2e3/Tet2 pathway is essential to the antidepressant effect of AZA, Tet2 conditional knockout (Tet2 cKO) mice were treated with AZA. Although Tet2 cKO mice exhibited longer immobility time in FST and TST compared with wild‐type littermate (Figure 9E,F), AZA did not reduce the immobility time of Tet2 cKO mice in FST and TST (Figure 9G,H), suggesting that the antidepressant effect of AZA was depended on the Nr2e3/Tet2 signaling pathway.

## 3 Discussion

Nr2e3 is the nuclear receptor family class 2, subfamily E, member 3, and is also named photoreceptor‐specific nuclear receptor gene (PNR).^[^ ^24^ ^]^ Like other nuclear receptors, Nr2e3 contains a DNA binding domain at the C‐terminal and a ligand binding domain at the N‐terminal.^[^ ^25^ ^]^ Nr2e3 directly binds to the promoter region of the gene through the DNA binding domain and regulates the transcription.^[^ ^26^ ^]^ Nr2e3 is abundant in rod cells of the retina and is located in the nucleus of rod cells. In the retina, Nr2e3 functions as a bi‐direction modulation factor and is a transcriptional activator of rod‐specific genes and transcriptional repressor of cone‐specific genes,^[^ ^15^ ^]^ therefore, Nr2e3 is a cell‐fate determination factor of the photoreceptor. Mutations of Nr2e3 are correlated with several hereditary retinopathies, such as enhanced short‐wavelength sensitive cone syndrome,^[^ ^27^ ^]^ retinal pigments,^[^ ^28^ ^]^ Goldmann–Favre syndrome.^[^ ^29^ ^]^ Except for the retina, Nr2e3 is also expressed in other tissues, including the breast and liver, and is related to breast and liver cancer. Estrogen receptor 1 (ESR1) is a key transcription factor for responding to tamoxifen treatment in ESR1‐positive breast cancer. Nr2e3 could activate the expression of ESR1 through binding to the promoter region of ESR1, and upregulation of Nr2e3 is related to recurrence‐free survival of ESR1‐positive breast cancer after tamoxifen treatment.^[^ ^30^ ^]^ Meanwhile, Nr2e3 also promotes the metastasis of ESR1‐negative breast cancer through upregulation of the expression of IL13α2.^[^ ^31^ ^]^ In addition, Nr2e3 reduction is also involved in the poor prognosis of liver cancer.^[^ ^32^ ^]^ However, whether Nr2e3 is expressed in the central nervous system and involved in the epigenetic mechanism of depression is unknown.

### 3.1 Nr2e3 is a Critical Transcription Factor of Tet2 in Depression

Epigenetic modification is a production of gene‐environment cross‐talk, epigenetic mechanism is a critical cause of major depressive disorder and chronic stress exposure.^[^ ^33^, ^34^ ^]^ As a DNA hydroxymethylase, Tet2 has been reported to be involved in the pathogenesis of various neurological diseases, such as ischemic stroke,^[^ ^35^ ^]^ multiple sclerosis,^[^ ^36^ ^]^ Alzheimer's disease,^[^ ^37^ ^]^ and Parkinson's disease.^[^ ^38^ ^]^ Our team focuses on the role of the TET enzymes, particularly Tet2, in the mechanism of depression. Our previous studies have demonstrated that conditional knockout of Tet2 in the nervous system or knockout of Tet2 specifically in the hippocampus of adult mice can induce depression‐like behaviors,^[^ ^8^, ^11^ ^]^ suggesting that Tet2 is a key epigenetic factor mediating depression‐like behaviors in mice. Although recent studies have investigated the downstream signaling pathway of Tet2,^[^ ^5^ ^]^ and the transcriptional regulation of Tet2 remains unclear. In this study, we identified Nr2e3 as a novel transcriptional regulator of Tet2 expression in depression. We found that Nr2e3 bound to the promoter region of Tet2 directly, increased the transcriptional activity of the Tet2 promoter, and enhanced the expression of Tet2. These findings suggest that Nr2e3 is a transcriptional activator of Tet2. However, one limitation of this study is that the transfection of a promoter–reporter plasmid may not accurately mimic the endogenous chromatin structure of the Tet2 gene. This is because the long sequence of the full‐length Tet2 gene makes it difficult to construct suitable expression plasmids.

Nr2e3 is a well‐documented transcriptional factor in the retina.^[^ ^39^ ^]^ During the development of the retina, Nr2e3 plays a critical role as a fate‐determine factor of the photoreceptor, while Nr2e3 is also required for the maintenance steady state of the mature retina.^[^ ^16^ ^]^ In rod photoreceptor cells, Nr2e3 interacts with other co‐regulators, to promote rod gene expression, while suppressing cone gene expression, such as Nrl, Nr1D1, and Crx.^[^ ^15^ ^]^ Interestingly, we also identified that DHX30 and LSD1 are co‐regulators of Nr2e3 under stress conditions. DHX30 and Nr2e3 synergistically promoted the expression of Tet2; meanwhile, LSD1 partially antagonized the increase of Tet2 by Nr2e3. These results indicate that DHX30 is a transcription coactivator of Nr2e3 and LSD1 is a transcription corepressor of Nr2e3 under stress.

### 3.2 Nr2e3 Has an Antidepressant Role in Mice

Although most previous studies focused on the functional study of Nr2e3 on the retina,^[^ ^40^ ^]^ recent studies have also reported that unfulfilled/DHR51(UNF), the ortholog of Nr2e3 in drosophila, exits in mushroom body neurons and a few central nervous system cells.^[^ ^41^ ^]^ UNF mutant is related to neural defects, such as compromised fertility and wing expansion failure.^[^ ^41^ ^]^ UNF also enhanced neuronal re‐extension during development.^[^ ^42^ ^]^ These results indicated that Nr2e3 was also involved in the central never system. In this study, we found that knockdown of Nr2e3 in the hippocampus caused significantly depression‐like behaviors, suggesting that Nr2e3 had an antidepressant effect in the central never system.

Synaptic plasticity is the process of synaptic pruning and axonal regeneration in an activity‐dependent manner.^[^ ^43^ ^]^ Synaptic plasticity is the ability of the brain to eliminate redundant synapses or form new synapses.^[^ ^44^ ^]^ Increasing evidence suggests that depression is related to abnormal synaptic plasticity.^[^ ^45^ ^]^ Brain‐imaging studies on patients with major depressive disorder have shown that there is abnormal synaptic plasticity in different brain regions, such as the hippocampus and prefrontal cortex.^[^ ^46^, ^47^ ^]^ Animal model studies have also demonstrated that stress can lead to a reduction of dendritic branches and synaptic plasticity.^[^ ^48^ ^]^ Synaptic plasticity is regulated by many signaling pathways, such as Akt‐mTOR signaling and brain‐derived neurotrophic factor signaling.^[^ ^49^, ^50^, ^51^ ^]^ In our study, we found significant impairment of synaptic plasticity in hippocampal neurons of Nr2e3KD mice, including the reduction of dendritic spines, accompanied by the downregulation of PSD95, Syn‐1 and NMDAR1. PSD95 has been identified as a scaffolding protein to recruit NMDAR and other signal pathway proteins.^[^ ^52^ ^]^ Mechanically, the silence of Nr2e3 may reduce the expression of Tet2, then change the 5hmC modification of genomic DNA, and decrease the expression of synaptic‐related proteins, resulting in the impairment of synaptic plasticity. Therefore, our results indicate that Nr2e3/Tet2 is a novel signaling pathway to regulate synaptic plasticity in stress‐related conditions.

### 3.3 Enhanced Nr2e3/Tet2 Signaling Pathway Exhibits Antidepressant Activities

Fast‐onset antidepressants represent one of the most urgent medical needs, particularly for patients with major depressive disorder.^[^ ^53^ ^]^ Currently, available antidepressants function by increasing the level of 5‐hydroxytryptamine (5‐HT), including 5‐HT receptor antagonists, 5‐HT reuptake inhibitors, monoamine enzyme inhibitors, and heterocyclic compounds.^[^ ^54^ ^]^ These drugs provide relief only in 60% of patients, have extensive side effects, and take 3 to 4 weeks to improve depressive symptoms.^[^ ^55^ ^]^ Recently, ketamine has been reported as a rapid antidepressant, however, ketamine is also addictive, and its abuse potential is also a major concern.^[^ ^56^ ^]^ ZZL‐7 has been identified as a fast‐onset antidepressant, responds at 2 h after administrated, and is well tolerated,^[^ ^57^ ^]^ but more research is needed. In this study, the results showed that AZA improved depression‐like behaviors within 1‐week after AZA administration. The treatment with AZA significantly increased the expression of PSD95 in stressed mice and improved synaptic plasticity. Previous studies demonstrated that AZA was well tolerated,^[^ ^58^ ^]^ therefore, AZA may be a potential fast‐onset antidepressant that needs more investigations.

## 4 Conclusion

The present study identified Nr2e3 as an essential transcription factor of Tet2, elucidated the upstream regulatory mechanism of Tet2, and clarified a new mechanism of depression by which Nr2e3 is involved in the regulation of synaptic plasticity through regulating Tet2. Our study demonstrated that Nr2e3 is a promising target for the development of novel antidepressant therapy and may be an early biological marker for depression. Our study indicated that regulating epigenetic modifications is an effective approach and important strategy for treating depression; importantly, our findings have important theoretical and clinical translational value for the development of new drugs for depressive disorders and social defects.

## 5 Experimental Section

### Animals

All the animal experiments were approved by the University Committee on Animal Care of Soochow University, in accordance with the National Institutes of Health animal operation guidelines, and all animals were maintained under specific‐pathogen‐free conditions in the animal facility of Soochow University. Male C57BL/6 mice were purchased from SLAC Company (Shanghai, China). *Tet2^flox/flox^* line (stock number 017573, B6; 129S4‐Tet2tm1.1Iaai/J) and Nestin‐Cre mice (stock number 003771, B6. Cg‐Tg (Nes‐cre) 1Kln/J) were purchased from the Jackson Laboratory (MA, USA), we obtained Tet2 nestin‐conditional knockout (Tet2 cKO) mice through mouse mating. Mice were housed under standard laboratory conditions (22 ± 1 °C, 60% humidity, 12 h light–dark cycle with lights on at 7:00 a.m.). 6 to 8‐week‐old male mice were used in all experiments.

### CMS

Male C57BL/6 mice were exposed to two types of environmental stressors daily. The stressors were different and randomized. The stressors were described as follows: wet cage and 30° cage tilt along the vertical axis for 24 h, inverted light and dark for 24 h, empty cage, and food deprivation for 24 h, water deprivation for 24 h, spatial restraint stress for 2 h, noise for 15 min, cage change for 24 h, and forced swimming at 10 °C for 5 min.

### Stereotactic Injection of AAV Virus

C57BL/6 mice were analgesized by intraperitoneal injection (ketoprofen, 5 mg kg^−1^), body temperature was maintained on a heating pad and artificial tears were applied to prevent eye drying. C57BL/6 mice were microinjected with AAV2/9‐RNAi control (titer: 1.08E + 13 v.g mL^−1^, Shanghai Genechem, China) and AAV2/9‐Nr2e3‐RNAi (titer: 4.25E + 13 v.g mL^−1^ Shanghai Genechem, China). Nr2e3 RNAi sequence was 5′‐CCAAGATGCTGTGCAGAAT‐3′. All the AAV‐virus was diluted to 1 × 10^12^ v.g mL^−1^ and was injected bilaterally into the hippocampus at 1 µL per site. The microinjection coordinates were 2.5 mm posterior to the Bregma, ±2.0 mm lateral to the sagittal midline, and at a depth of 2.0 mm from the cranial surface. The needle was left in the tissues for 10 min before the withdrawal. Behavioral tests were performed at 21 days post‐AAV microinjection.

### AZA Administration

AZA (S3196, Selleck Shanghai, China) was dissolved in DMSO (53 mg mL^−1^) and diluted with normal saline. According to the literature,^[^ ^59^ ^]^ 5 mg kg^−1^ was selected as the dose of intraperitoneal administration in mice. Mice were injected intraperitoneally with one dose (5 mg kg^−1^ body weight) per day for 4 weeks. Control mice were injected with the same volume of normal saline.

### TST

TST was performed as described in the previous study.^[^ ^8^ ^]^ Mice were suspended on the edge of the desk from their tails (50 cm away from the floor). The immobility time of mice was recorded in 6 min. A double‐blind method was used in the experiment.

### FST

FST was performed as described previously.^[^ ^8^ ^]^ The mouse was placed in a 2‐L beaker containing 1.2–1.6 L of water. Water temperature was maintained at 21–24 °C. After the mouse was transferred into the water, the immobility time of the mice was recorded for 6 min. Fresh water was replaced before another mouse was tested. A double‐blind method was used in this experiment.

### NSFT

NSFT was performed according to a previous study.^[^ ^11^ ^]^ This test was performed in a square open field box (45 × 45 × 40 cm^3^, white color). Prior to the experiment, the mice were fasted for 24 h. A single pellet of food was put in the center of the box, and the time started to record when the subject mouse was placed in the corner of the box back to food, the record was stopped when the mice started to eat food. The test lasts for 5–10 min.

### OFT

An open‐field test was performed according to the previous studies.^[^ ^60^ ^]^ The test was performed in a square open field box (40 × 40 × 40 cm^3^, white color). The top of the box was uncovered. The box was divided into outer and central zones, and the central zone was 20 × 20 cm^2^. The mouse was placed in the center of the box, and its movement trajectory was tracked within 10 min by an ANY‐maze system. The speed, time, and traveling distance of the mouse in the outer/central zone were analyzed. The surface of the box was cleaned with 75% ethanol before another mouse was tested.

### Western Blot

Tissues or cells were harvested and lysed using the RIPA lysis buffer containing 0.5% sodium deoxycholate, 0.1% SDS, 1% Triton X‐100, 1 mm EGTA pH 8.0, 1 mm EDTA pH 8.0, 150 mm NaCl, 50 mm Tris pH 8.0, and 1×protease inhibitor cocktail. The mixture was incubated on ice for 30 min and then centrifuged at 12000×g at 4 °C for 15 min. The protein suspension was collected and the concentration was determined using a BCA kit. The protein sample was boiled with the loading buffer containing dithiothreitol for 10 min. Proteins were separated by 8% or 10% sodium dodecyl sulfate‐polyacrylamide gel and electro‐transferred to PVDF membranes using a transfer system (Bio‐Rad, California, USA). The membranes were blocked with 5% non‐fat milk for 1 h at RT and then were incubated with primary antibodies overnight at 4 °C. The primary antibodies were diluted as follows: anti‐Nr2e3 (1:200; Santa Cruz, CA, USA), anti‐Tet2 (1:1000, Abcam, UK), anti‐GAPDH (1:10000, Santa Cruz, CA, USA), anti‐actin (1:10000, Santa Cruz, CA, USA), anti‐tubulin (1:10000, Santa Cruz, CA, USA), anti‐PSD95 (1:1000, Abcam, UK), anti‐Syn1 (1:1000, Abcam, UK), anti‐NMDAR1 (1:200, Santa Cruz, CA, USA), anti‐flag (1:3000, Sigma, USA). After washing three times with PBST, the membranes were incubated with the anti‐mouse or anti‐rabbit horseradish peroxidase‐conjugated secondary antibodies at room temperature for 1 h and visualized by immobilon Western Chemiluminescent HRP Substrate ‐(ECL). The quantification of band densities was analyzed with image lab software (Bio‐Rad, USA).

### Dot Blot

Total genomic DNA was extracted by using the phenol‐chloroform method and denatured by treatment with 2 n NaOH. DNA was spotted onto a nitrocellulose membrane, dried at room temperature for 20 min, and then baked at 80 °C for 30 min.

The membrane was blocked with 5% non‐fat milk for 1 h at room temperature, and incubated with an anti‐5hmC antibody (1:10000, Active Motif, USA) at 4 °C overnight. The membrane was washed with PBST and then incubated with horseradish peroxidase‐conjugated anti‐rabbit antibody for 1 h at room temperature. The 5hmC signal was visualized using immobilon Western Chemiluminescent HRP Substrate (ECL). Quantification of band densities was analyzed using image lab software (Bio‐Rad, USA).

### qPCR

Total RNA was extracted from cells and tissues by using Trizol regents (DP424, TIAGEN, China). The cDNA was obtained using ABScript III RT Master Mix for quantitative PCR with gDNA Remover according to the protocol. Briefly, 1 µg of total RNA was reversed to cDNA in a 20‐µL system (Abclonal, Wuhan, China). Quantitative PCR was performed using 2×Universal SYBR Green (Abclonal, Wuhan, China) on the Step one plus Real‐Time PCR system (Applied Biosystems, Carlsbad, CA, USA). The relative mRNA level of the target gene was calculated by using the 2^−ΔΔCT^ method and normalized to GAPDH. Primers for the target genes were listed in Table S1 (Supporting Information).

### Plasmids Constructions

The full‐length of mouse Tet2 promoter luciferase reporter plasmid (pGL3‐Tet2 promoter F) was purchased from Addgene (63879, Addgene, MA, USA). The truncated Tet2 promoter constructs containing different Nr2e3 binding motifs were amplified from pGL3‐Tet2 promoter F by PCR and were sub‐cloned into the pGL3‐Basic at the MluI/XhoI sites using 2 × MultiF Seamless Assembly Mix (RK21020, Abclonal, China). The primers are listed in Table S2 (Supporting Information). The coding sequence (CDS) of Nr2e3, Nrl, CRX, LSD1, DHX30, NR1D1, Sin3A, and NCOA1/2/3 were amplified from mouse cDNA and then sub‐cloned into the pCDNA3.1‐myc‐hisC at BamHI/XhoI sites using 2 × MultiF Seamless Assembly Mix. Primers were listed in Table S3 (Supporting Information).

### Cell Culture and Plasmid Transfection

The hippocampal neuronal cell line HT‐22, Neuron 2a cell (CCL‐131, ATCC), and 293T cell were cultured in DMEM (Hyclone, UT, USA) supplemented with 10% fetal bovine serum (Gibco, CA, USA) at 37 °C, 5% CO_2_. Plasmid was transfected using PEI‐MAXTransfection (Polysciences, PA, USA) when cells reached 80–90% confluence. The plasmid was diluted in Opti‐MEM (Gibco, CA, USA) (50:1 of volume to µg plasmid), and the PEI (3:1 of volume to µg plasmid) was also diluted with Opti‐MEM (50:1 of volume to µg plasmid). The PEI mixture was then added to the plasmid, gently mixed, incubated at room temperature for 20 min, added dropwise to the cell, and swirled the plate immediately. Cells were incubated at 37 °C with 5% CO_2_ and the expression of the target gene was detected at 48 h post‐transfection.

### siRNA Transfection

Neuro‐2a cells were seeded in the 12‐well plate 24 h before transfection, and siRNA was dissolved in RNase‐free H_2_O at 20 µm. 2.5 µL Nr2e3 siRNA and 2 µL of Lipo3000 were diluted in 50 µL opti‐MEM individually, and then Lipo3000 was added into siRNA, gently mixed, and then incubated at RT for 20 min. The mixture was added dropwise to the cell and the cell was harvested 48 h after transfection to detect the mRNA levels of Nr2e3 and Tet2 by using qPCR. The sequences of two Nr2e3 siRNA were siRNA‐1: 5′‐ccaagatgctgtgcagaat‐3′ and siRNA‐2: 5′‐ccgccgaaacttgtgctaa‐3′.

### Construction of HT22 Nr2e3 Overexpression Stable Cell Line

The lentivirus of Nr2e3 overexpression was packaged through co‐transfection of lenti‐CRIPSR‐v2‐Nr2e3‐3×Flag plasmid with two helper plasmids into 293T cells. The ratio of pMD2.G: psPAX2: plenti‐Nr2e3‐3×Flag is 5:8:7. After 48 h of transfection, the suspension was harvested, and 1/5 PEG8000/NaCl was added into the suspension. They were mixed completely at 4 °C overnight. The viral supernatant was centrifuged at 8000 rpm for 30 min to pellet the virus and then diluted in the fresh medium. The HT22 cell line was infected by Nr2e3‐overexpression lentivirus for 12 h, and the polybrene (TR1003, Sigma–Aldrich, USA) was added at a final concentration of 5 µg mL^−1^. After 12 h, the fresh medium was replaced, and the cell was incubated for 48 h. The puromycin (2 µg mL^−1^, 58‐58‐2, Solarbio, Beijing, China) was added to screen the positive clones. After the screening, the puromycin‐positive clones were subcloned and a single clone was selected to further test the expression of Nr2e3.

### Construction of Nr2e3 Knockout HT‐22 Cell Line Based on CRISPR‐Cas9

The single guide (sgRNA) was designed by using benchling software online. Two sgRNAs respectively target exon2 and exon3 were selected, sgRNA1: tcgctccagtgccgagtgtg; sgRNA2: agtggataaggcccatcgca. The primer sequences for construction were sgRNA1 (forward) 5′‐CACCGtcgctccagtgccgagtgtg‐3′ (reverse) 5′‐aaaccacactcggcactggagcgaC‐3′; sgRNA2 (forward) 5′‐ aaacagtggataaggcccatcgcaC‐3′ (reverse) 5′‐CACCGtgcgatgggccttatccact‐3′. lenti‐CRISPR v2 (addgene plasmid: 52960) was digested with BsmBI (ER0451, Thermo Fisher, USA). The sgRNA was annealed and inserted into lenti‐CRISPR v2. The lentivirus was packaged through co‐transfecting lenti‐CRIPSR‐v2‐Nr2e3‐sgRNA with two helper plasmids into the 293T cell line. The ratio of pMD2.G: psPAX2: lenti‐CRISPR v2‐Nr2e3 sgRNA is 5:8:7. The HT22 cells were infected with lentivirus. After 48 h of infection, the cells were screened with puromycin at a concentration of 2 µg mL^−1^ for 1 week, and then single clone cells were generated by limiting dilution into 96‐well plates. The clones were digested using trypsin and the genome DNA was extracted. The production containing the sgRNA binding site was amplified by touchdown PCR and was subsequently digested with T7E1 (T7 EndonucleaseI, NEB#E3321, New England Biolabs, MA, USA), finally analyzed on 2% agarose gel. Mutant production was further analyzed through DNA sequencing. The primers for touchdown PCR were sgRNA1(forward) 5′‐cagcaacttctagcaagcag‐3′ (reverse) 5′‐tccttctcacactcctcttg‐3′; sgRNA2(forward) 5′‐ tgggagtcacatgggcagat‐3′ (reverse) 5′‐tgtttccatggcatccaggt‐3′. The clones with a mutation in Nr2e3 DNA were selected for further research.

### Dual‐Luciferase Reporter Assay

HT22 Nr2e3KO cells or 293T cells were seeded in the 24‐well plate at 24 h before transfection. Once the cells reached 80–90% confluency, pGL3‐basic‐Tet2 promoter constructs were transfected with the Nr2e3 or other gene overexpression plasmid, and pRL‐TK (D2760, Beyotime, China) was also transfected to normalize the transfection efficiency. The ratio of promoter: overexpression plasmid: pRT‐TK was 100:100:1. The total of 1 µg plasmid was transfected into one well using PEI. The cells were harvested 48 h post‐transfection and the luciferase activity was measured by using a Dual‐Luciferase reporter assay kit (DL101‐01, Vazyme, China).

### CHIP Assay

The Chip assay was performed on the hippocampal tissues of CMS mice as described previously.^[^ ^61^ ^]^ The tissue was cut into 1‐mm pieces and suspended in 10 mL PBS. Subsequently, the tissue was cross‐linked by 5% formaldehyde and was shanked for 10 min at room temperature. 100 µL of 1.375 m glycine per mL was added to quench the cross‐link. The tissue was ground in liquid nitrogen, washed with PBS, and then lysed in CHIP lysis buffer containing a 1×protease inhibitor cocktail (B14001, Biomaker, USA). The genomic DNA was extracted by using the phenol‐chloroform method. Subsequently, the chromatin DNA was fragmented to 200–500 bp by sonication at 10% amplitude for 20 min on an ice–water mixture, and each pulse consisted of 3 s of sonication followed by a 5 s rest. Immunoprecipitation was performed as described in the previous study.^[^ ^62^ ^]^ Briefly, the lysate and anti‐Nr2e3 antibody (sc‐374513, Santa Cruz, USA) was mixed and rotated overnight at 4 °C. The mass ratio of DNA to anti‐Nr2e3 was 5:1. The protein A/G magnetic beads were activated using CHIP lysis buffer, and were blocked by 2% BSA and 100 µg mL^−1^ herring sperm DNA (D3159, Sigma–Aldrich, USA) for 1 h at room temperature. Subsequently, the mixture of anti‐Nr2e3 antibody and DNA was further incubated with blocked protein A/G for 1.5 h at 4 °C. The beads were washed with high‐salt wash buffer, low‐salt wash buffer, and LiCl wash buffer, and then eluted using elution buffer. To extract the DNA, 1 µL of protease K (2 µg µL^−1^) and 4.8 µL 5 m NaCl were added to the productions and reversed cross‐linked at 65 °C overnight, and then DNA was extracted using Phenol‐chloroform. The enrichment of the Tet2 promoter region was detected by using qPCR. The primers are listed in Table S4 (Supporting Information).

The cell CHIP‐PCR was performed as described previously.^[^ ^63^ ^]^ In brief, pCDNA3.0‐Nr2e3 and pGL3‐Tet2 promoter F (#63879, addgene, MA, USA) were co‐transfected into 293T cells. After 48 h, the production of CHIP was used to detect different Tet2 promoter fragments by PCR, the sequences of the primer Tet2‐10.3F and Tet2‐10.3R were listed in Table S4 (Supporting Information).

### 5hmC‐Specific Chemical Labeling, Affinity Purification, and Sequencing

5hmC enrichment and capture were performed as previously described.^[^ ^64^ ^]^ 20–50 ng of input genomic DNA or 5hmC‐captured DNA were used for DNA library preparation, following the protocol of NEBNext Ultra II DNA Library Prep Kit for Illumina (E7645S, New England Biolabs, MA, USA) and NEBNext Multiplex Oligos for Illumina (E7600S, New England Biolabs, MA, USA). Illumina Hi‐seq 2000 machines were used for running sequencing libraries.

### Primary Neuron Culture

The pregnant C57 mice were anesthetized on day 15 of pregnancy and the abdominal skin was sterilized with 70% ethanol. The embryos were then collected from the uterus after the peritoneal cavity was opened. The embryos' brains were decapitated and the cerebral cortex was dissected under a stereo microscope before being placed into cold PBS on ice. The cerebral cortex was then cut with scissors and digested with 0.25% trypsin at 37 °C for 5 min, and the digestion was terminated with DMEM/F12 (SH30261.02, Hyclone, Utah, USA) +10% FBS (SH30406.05, Hyclone, Utah, USA) medium. The resulting cell suspension was filtered through a 40µm cell strainer, centrifuged, and then resuspended in 10 mL of primary neuronal culture medium (Neurobasal, A1371001, Gibco, 50 mL; Glutamax, 35050061, ThermoFisher, 0.5 mL; 1× B27, 10889038, Thermo Fisher, 0.5 mL). The cells were then counted, diluted to a concentration of 1 × 10^6^, and seeded in 1 mL per well into polylysine‐coated 12‐well plates. After 4 d, the cells were infected with AAV‐Nr2e3‐RNAi and AAV‐RNAi control virus for 12 h, and the medium was changed every 3 d. The cells were lysed with RIPA lysis buffer at 8 d post‐infection to extract the protein, which was then used for Western blot analysis.

### Golgi Staining and Spine Density Analysis

The Golgi‐Cox staining was performed according to the manufacturer's protocol of the FD Rapid Golgi Stain kit (PK401, FD NeuroTechnologies, Columbia MD, USA). Briefly, the mouse brain was immersed in solution A/B for 14 d at room temperature in the dark, followed by immersion in solution C for 7 d. The brain tissue was embedded in OCT at −80 °C, and then sliced into 100 µm sections using the frozen microtome. The tissue sections were mounted onto slides and immersed in solution D/E for 10 min. The slices were dehydrated in ethanol and cleared in xylene before covering using neutral resin. The individual neurons were photographed using a 40× objective (AXIO SCOPEA1, ZEISS). The dendritic spines were counted by an experimenter that blinded to the group of each group (*n* = 3).

### Small Compounds Administration

HT‐22 cells were treated with antidepressants and small compounds. The work concentration of the drug was selected at 10 µm according to the previous references^[^ ^65^ ^]^ and the company's recommendation. When the cells reached 30–40% confluency, the drugs were added to the medium. The cells were harvested to extract RNA after 24 h, 1 µg of RNA was reverse transcribed into cDNA using ABScriptIII RT master mix (RK20429, Abclonal, china), and the mRNA levels were determined through using qPCR.

### Statistical Analysis

All data were presented as the mean ± SEM. Graph Pad Prism Software (Version 6, La Jolla, CA) was used to analyze data. Student's *t*‐test was used to compare the differences between two groups, and one‐way or two‐way analysis of variance followed by Tukey's multiple comparison tests was used to analyze the differences among three or more groups. Detailed statistical analysis methods for each comparison were indicated in the figure legends, including sample size and data presentation. Statistical significance was set at *p* < 0.05.

## Conflict of Interest

The authors declare no conflict of interest.

## Author Contributions

X.X. and M.Z. designed experiments, M.X., X.S., Z.Y., Z.Q., Y.H., W.B., and Y.Y. performed the experiments, collected data, and analyzed data. M.X., M.Z., and X.X. wrote the manuscript.

## Supporting information

Supporting Information

## Data Availability

The data that support the findings of this study are available from the corresponding author upon reasonable request.
