# Supplementary material for: Targeting Nr2e3 to Modulate Tet2 Expression: Therapeutic Potential for Depression Treatment
Source: Adv Sci (Weinh). 2024 Jun 17;11(31):2400726. doi: 10.1002/advs.202400726 (PMC11336902; doi:10.1002/advs.202400726)
Supplement: Supplementary file 1 — Supporting Information [file ADVS-11-2400726-s001.docx]

**Supplemental data**

**
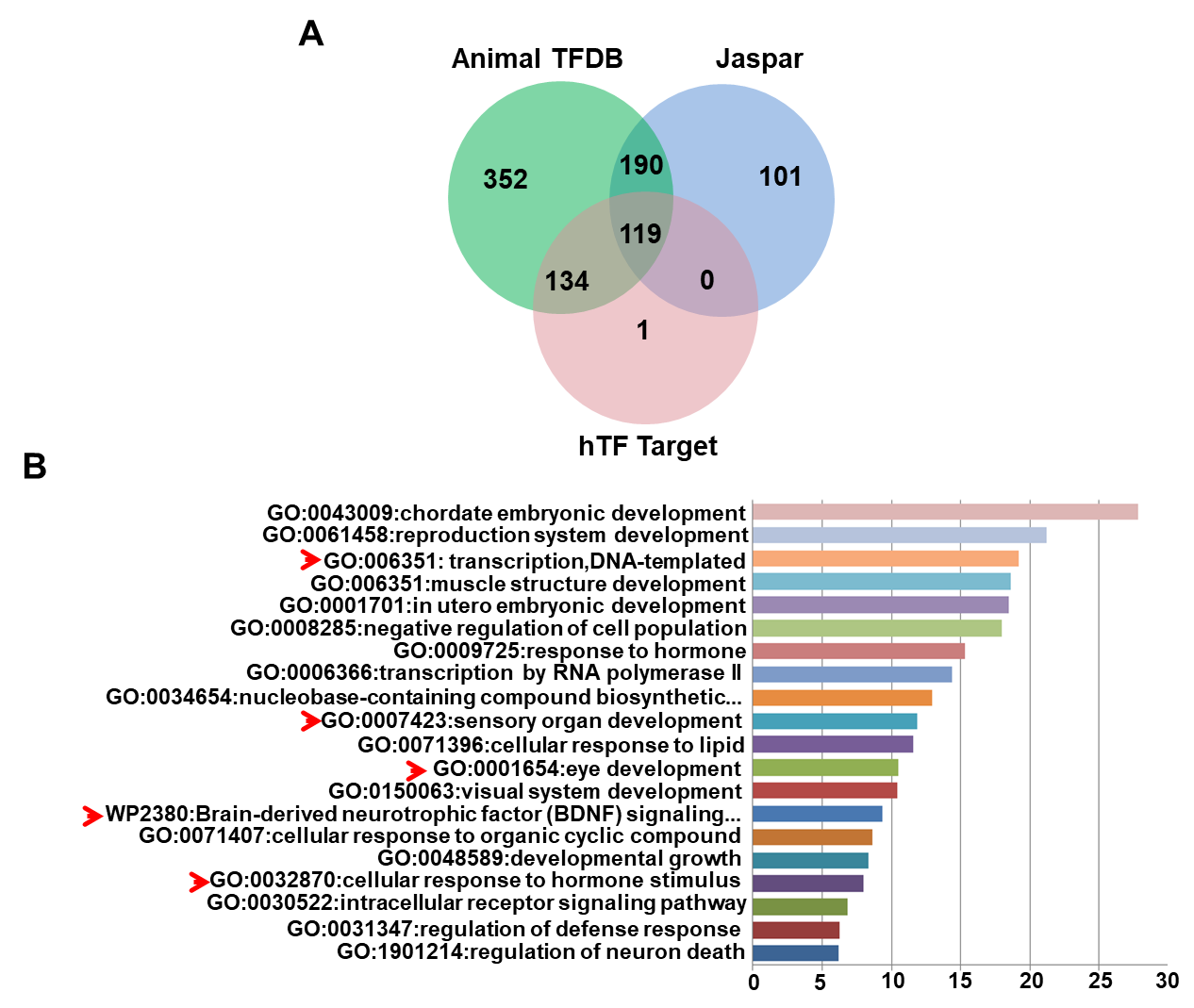
**

**Fig. S1. Transcription factors of Tet2 were selected by different software.** A. The diagram showed 119 potential transcriptional factors of Tet2, which have been identified through overlapping of predictive transcription factors by three software including Jaspar, animal TFDB, and hTF target. B. Gene ontology (GO) analysis were performed on total 119 potential transcriptional factors of Tet2.

**
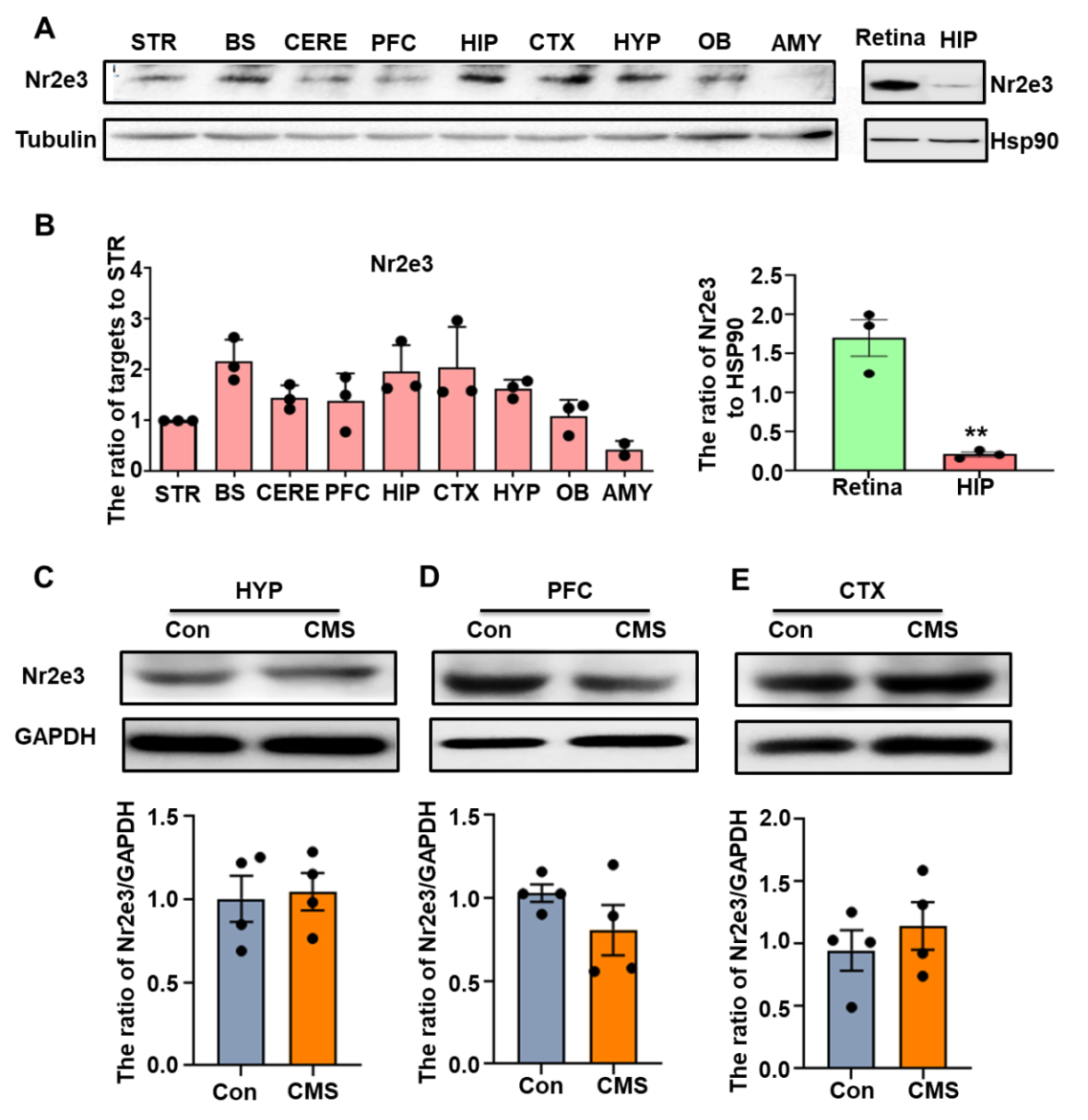
**

**Fig. S2. Nr2e3 was distributed in the different mouse brain regions.** A. Brain tissues including the striatum (STR), the brainstem (BS), the cerebellum (CERE), the prefrontal cortex (PFC), the hippocampus (HIP), the cortex (CTX), the hypothalamus (HYP), the olfactory bulb (OB), and the amygdala (AMY) were collected for Western blot. Protein levels of Nr2e3 were examined in different brain regions. Nr2e3 protein in the retina was also examined as a positive control. Protein levels of Nr2e3 were quantitatively analyzed in different brain regions (n=3). C-E. Mice were treated different stressors daily to induce CMS mice. The brain tissues including HYP (C), PFC (D) and CTX (E) were collected. Protein levels of Nr2e3 were examined by Western blot analysis (n=4). All data were presented as mean ± SEM. Statistical significance was determined by using Student’s t-test (**P < 0.01).

**
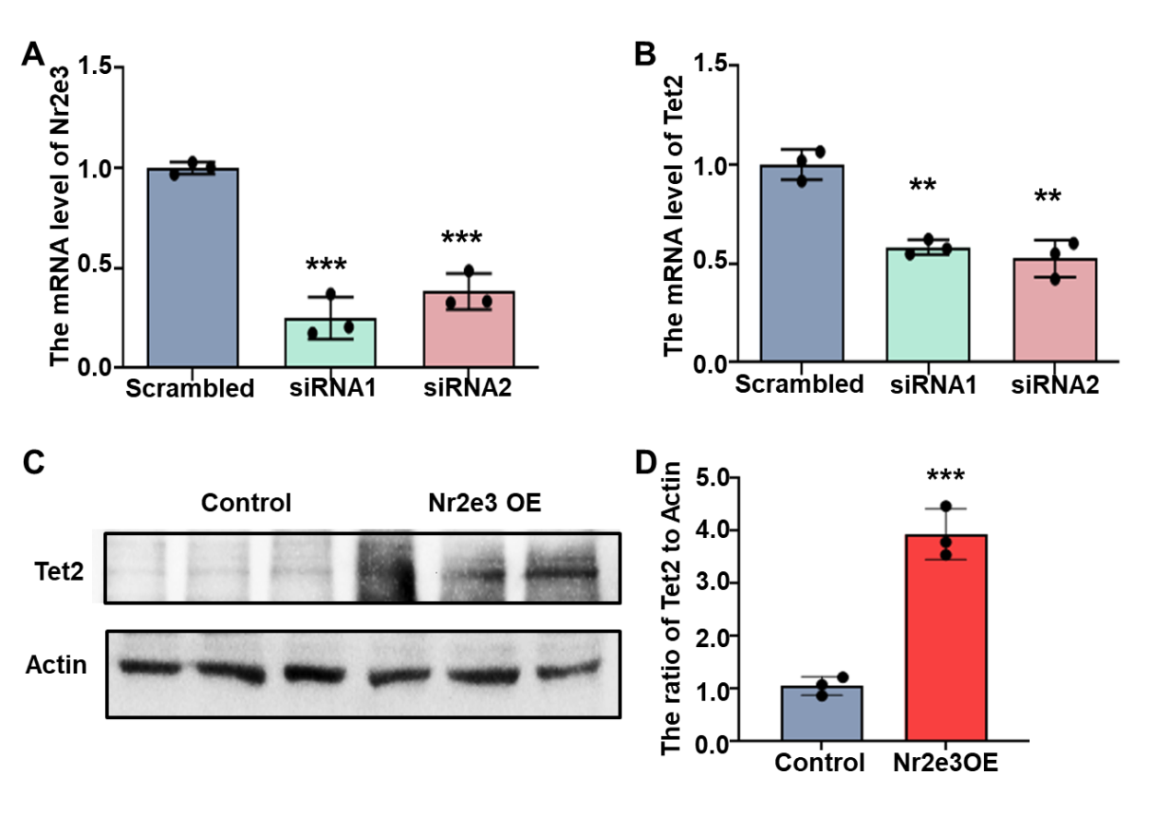
**

**Fig. S3.** **The expression of Tet2 decreased after the knockdown of Nr2e3 by siRNA and increased after Nr2e3 viral infection.** A. Neuron-2a cells were transfected with scrambled siRNA or Nr2e3 siRNA for 48 h. Then the cells were harvested and total mRNA was extracted at 48 h post transfection. Nr2e3 mRNA level was determined by quantitative PCR analysis (n=3). B. Similarly, Tet2 mRNA level was examined by quantitative PCR analysis in Nr2e3 siRNA transfected Neuron-2a cells (n=3). C. Tet2 protein level in Nr2e3OE cells was determined by using Western blot. D. Quantitative analysis of Tet2 protein level was performed (n=3). All data were presented as mean ± SEM. A, B by using one-way ANOVA with Tukey’s multiple comparison tests. D by using Student’s t-test (**P < 0.01 and ***P< 0.001).

**
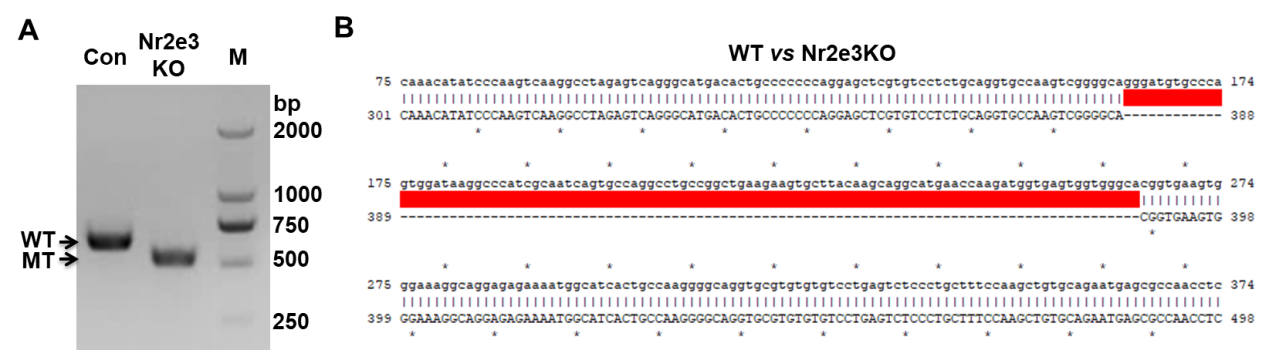
**

**Fig. S4.** **Nr2e3 KO cell line was constructed.** A. HT22 cells were used to produce a Nr2e3 KO cell line by CRISPR-Cas9. Base deletion in the Nr2e3 gene was confirmed by PCR analysis. B. DNA sequencing by the sanger method confirmed the base deletion by CRISPR-Cas9. Red part showed deleted bases in Nr2e3 KO cells.

**
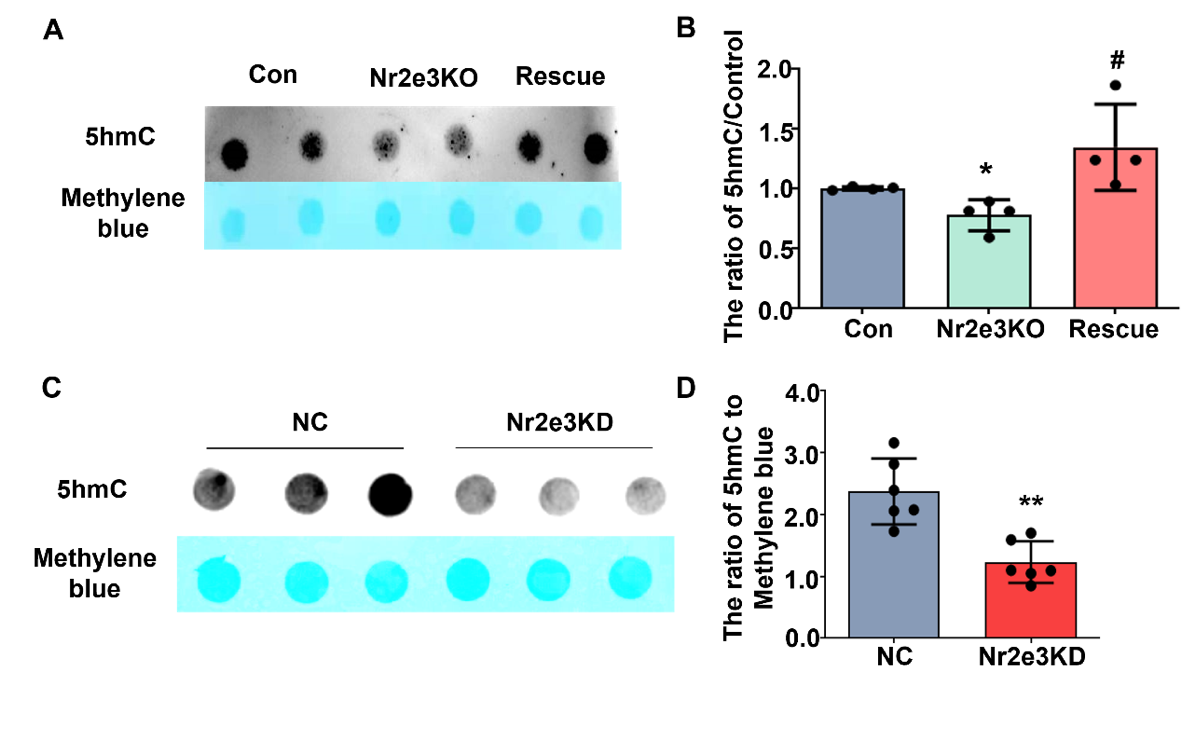
**

**Fig. S5. Nr2e3 affected 5hmC modification levels.** A. Dot blot was used to detect levels of DNA 5hmC in normal HT22 cells (Con), HT22 Nr2e3KO cells and HT22 Nr2e3KO cells infected with Nr2e3 lentivirus (rescue cells). B. Analysis of DNA 5hmC levels were performed (n=4). C. Genomic DNA was extracted from the hippocampus of Nr2e3 KD mice and negative control (NC) mice and subjected to Dot blot to examine DNA 5hmC levels. D. Quantitative analysis of hippocampal DNA 5hmC levels was performed (n=6). All data were presented as mean ± SEM. B by using two-way ANOVA with Tukey’s multiple comparison tests. D by using Student’s t-test. (*P < 0.05 and **P < 0.01 vs NC group or Con group; ^#^P < 0.05 vs Nr2e3KO group).

**
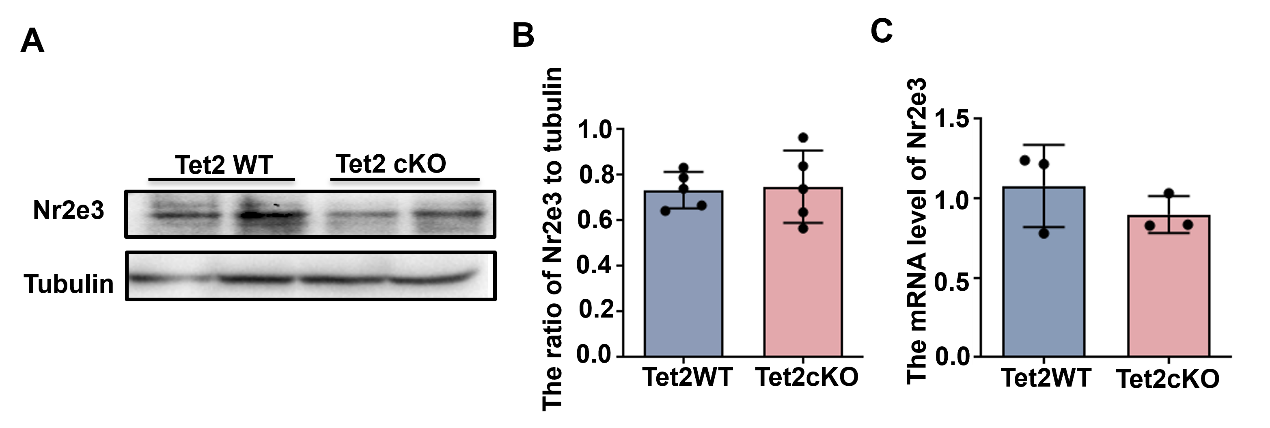
**

**Fig. S6.** **The expression of Nr2e3 was not changed in Tet2 cKO mice.** A. The hippocampal tissues of Tet2 nestin-conditional knockout (cKO) mice were collected. Protein levels of Nr2e3 were examined by Western blot. B. Protein levels of Nr2e3 were quantitatively analyzed (n=5). C. Nr2e3 mRNA level was also determined in the hippocampal tissue of Tet2 cKO mice (n=3). All data were presented as mean ± SEM. Statistical significance was determined by using Student’s t-test.


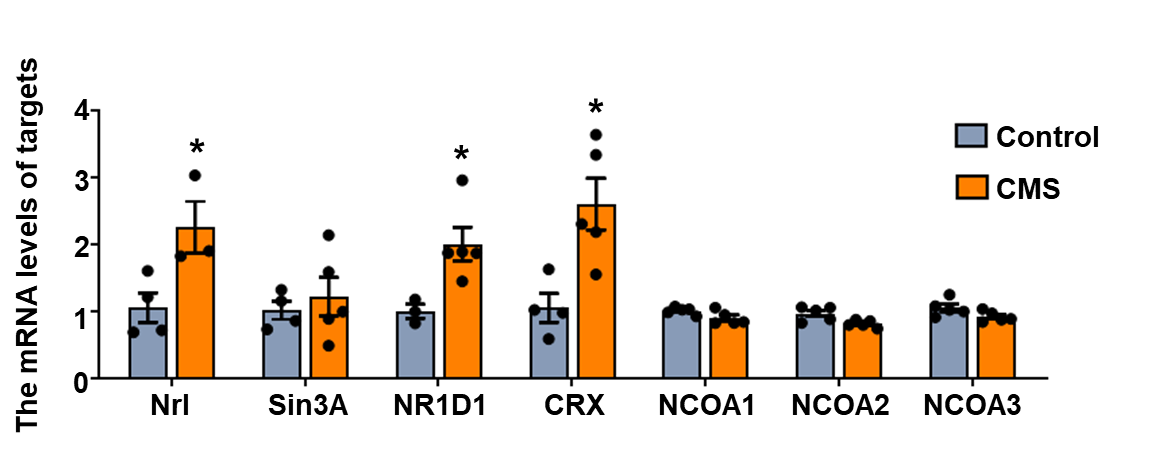


**Fig. S7.** **The expression of Nr2e3 co-regulators after stress were examined.** The hippocampus from CMS mice and control mice were collected and total RNA was extracted. The mRNA levels of different transcriptional factors were examined by quantitative PCR analysis (n=3-5). All data were presented as mean ± SEM. Statistical significance was determined by using two-way ANOVA with Tukey’s multiple comparison tests (*P < 0.05).


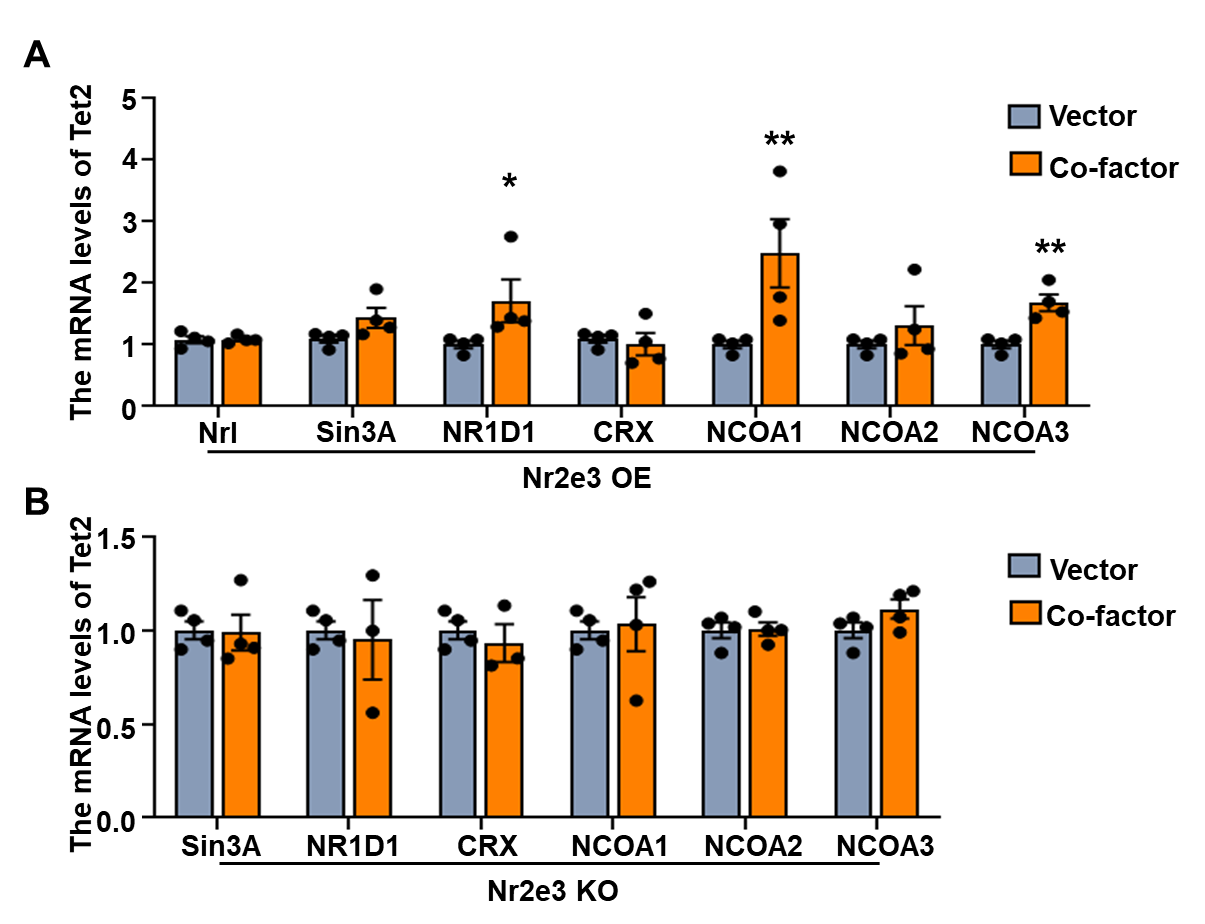


**Fig. S8.** **The levels of Tet2 mRNA were determined in HT22 Nr2e3OE and HT22 Nr2e3KO cells after transfection with Nr2e3 co-regulators.** A. HT22 Nr2e3OE cells were transfected with different Nr2e3 co-regulators and Tet2 mRNA levels were determined by quantitative PCR analysis at 48 h post transfection (n=4). B. HT22 Nr2e3KO cells were transfected with different Nr2e3 co-regulators. Tet2 mRNA levels were examined by quantitative PCR analysis at 48 h post transfection (n=3). All data were presented as mean ± SEM. Statistical significance was determined by using two-way ANOVA with Tukey’s multiple comparison tests (*P < 0.05 and **P < 0.01).


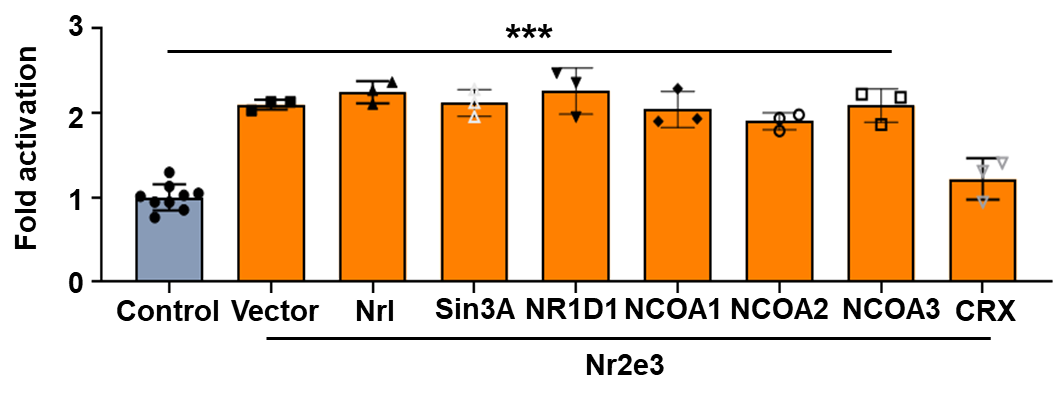


**Fig. S9. Dual-luciferase reporter assay was performed to detect Tet2 promoter activity.** Different Nr2e3 co-regulator plasmids were co-transfected with Nr2e3 plasmid into 293T cells. Tet2 promoter activity was detected using dual-luciferase reporter assay at 48 h after transfection (n=3). All data were presented as mean ± SEM. Statistical significance was determined by using one-way ANOVA with Tukey’s multiple comparison tests (***P< 0.001).


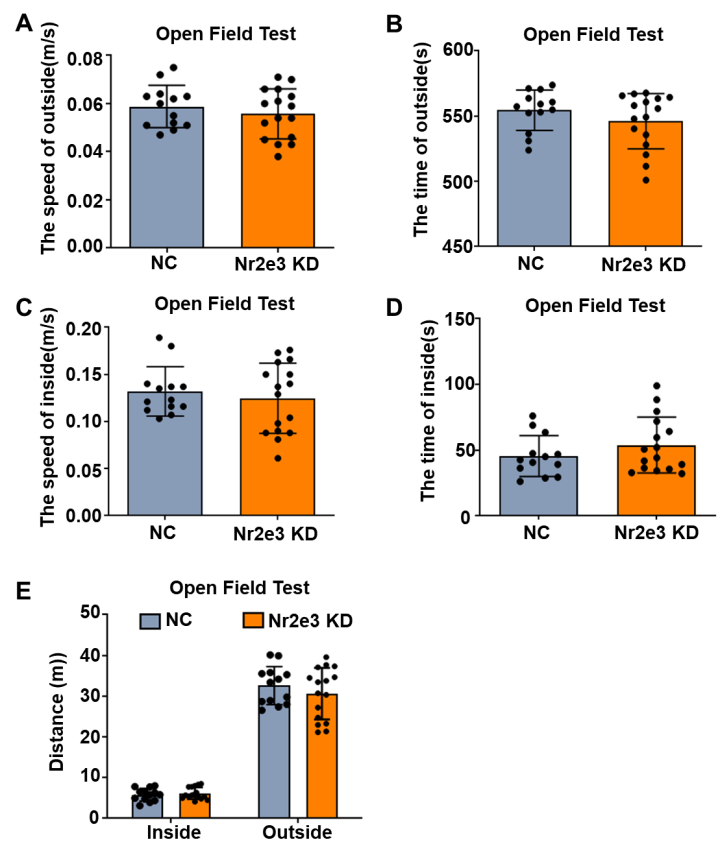


**Fig. S10. Open field tests in AAV-Nr2e3 RNAi-treated mice.** A. After mice were treated AAV-Nr2e3 RNAi or control AAV-RNAi for 21 d, open field tests were carried out. The walking speed of mice in the outside area was examined in two groups. B. The time stay in the outside area was record in two groups. C. The walking speed of mice in the central area was examined in two groups. D. The time stay in the central area was record in two groups. E. The distance in the central and outside areas was recorded N=13-16. All data were presented as mean ± SEM. A-D by using Student’s t-test. E by using two-way ANOVA with Tukey’s multiple comparison tests.

**
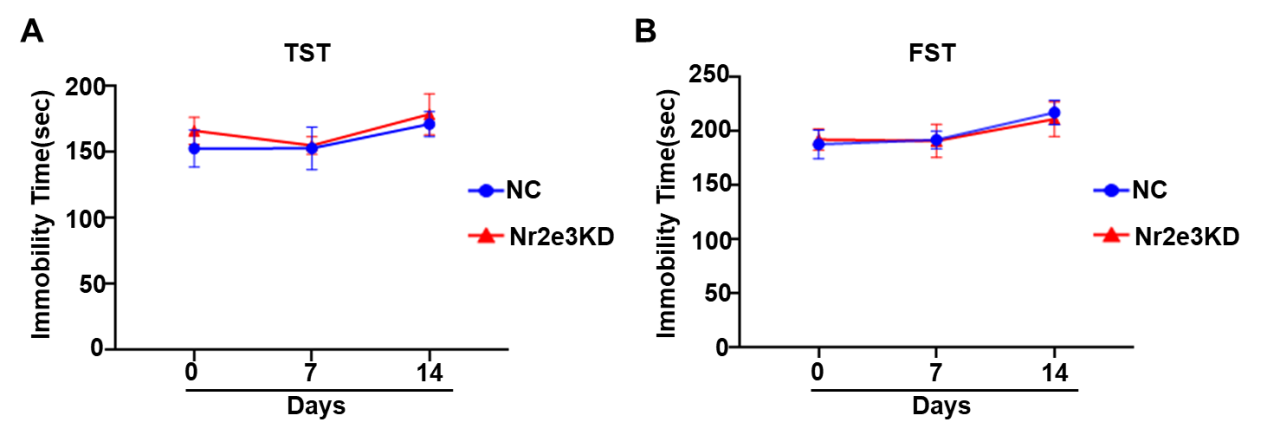
**

**Fig. S11. Knockdown of Nr2e3 in the hippocampus of Tet2 cKO mice did not alter depressive behaviors.** A-B. Following the injection of AAV-Nr2e3 RNAi virus into the hippocampus of Tet2 cKO mice, TST (A) and FST (B) were performed weekly (n=6). All data were presented as mean ± SEM. Statistical significance was determined by using two-way ANOVA with Tukey’s multiple comparison tests.

**
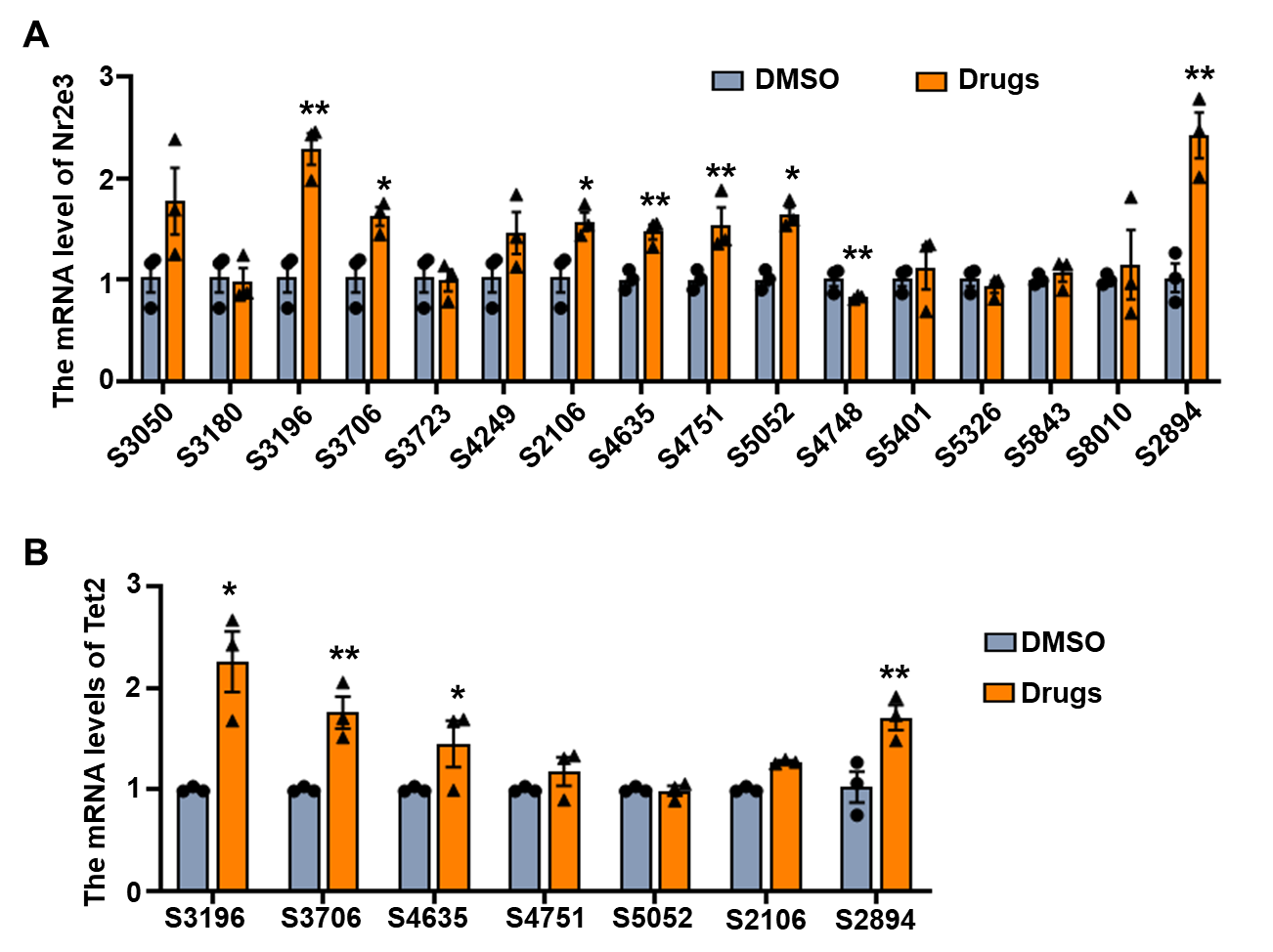
**

**Fig. S12. Drugs that increased Nr2e3/Tet2 expression was screened**. A-B. HT22 cells were treated with different small molecules (10 µM) for 24 h to screen drugs that can increase Nr2e3 expression. Nr2e3 (A) and Tet2 (B) mRNA levels were examined by quantitative PCR analysis (n=3). All data were presented as mean ± SEM. Statistical significance was determined by using two-way ANOVA with Tukey’s multiple comparison tests (*P < 0.05 and **P < 0.01).

**Supplemental Tables**

**Table S1**. The sequences of real-time PCR primers

| Gene Name | Sequence (5’→3’) |
| --- | --- |
| Tet2 F | TGTAAGTTTGCCAGAAGCAAG |
| Tet2 R | CTTCAGACCCAAACAGCAGT |
| LSD1 F | AAGCCAGGGATCGAGTAGGT |
| LSD1 R | GGAACAGCTTGTCCATTGGC |
| Pias3 F | CAGCTCAGATTCTGTCTCTGTG |
| Pias3 R | TTCTTGGTTGGAGGGAGGTAA |
| Nrl F | GTGACTACGTCATCTCTGCC |
| Nrl R | CAGAAGGCTCCCGCTTTATTTC |
| Nr2e3 F | GATGGCATCCATGAGACATC |
| Nr2e3 R | GCAGAGACCACTGTATGGCT |
| CRX F | GTTCAAGAATCGTAGGGCGAA |
| CRX R | TGAGATGCCCAAAGGATCTGT |
| Nr2e1 F | CTCCGGATTCTTCAAGAGGAG |
| Nr2e1 R | ACACTTCTTCAGTCGACACG |
| NR1D1 F | CTCCATCGTTCGCATCAATC |
| NR1D1 R | GGTTGTTGGCCAAGTTCATG |
| Sin3A F | CTGTCCTATCTTGACCAGGTGA |
| Sin3A R | GTGTTGTCACGTTCACCATG |
| DHX30 F | ACAGCGTGATTGGAAGAGCC |
| DHX30 R | CCTGACGCTCAGCATCAATC |
| mGAPDH F | CATGGCCTTCCGTGTTCCTA |
| mGAPDH R | GCCTGCTTCACCACCTTCTT |
| mNMDAR1 F | AGAGCCCGACCCTAAAAAGAA |
| mNMDAR1 R | CCCTCCTCCCTCTCAATAGC |
| NMDA2A F | GAACGCGAACTTCGAAATCTG |
| NMDA2A R | GTCAGTGCGGTTCATCAATAAC |
| NMDAR2B F | AAGAAGAATCGGAACAAACTGC |
| NMDAR2B R | CAGCTGGCATCTCAAACATATG |
| GluR1 F | GCAGCAGTGGAGGACAAGTGAC |
| GluR1 R | CCGCCATCACCTTCACACCATC |
| GluR2 F | AACAAATGGTGGTACGACAAAG |
| GluR2 R | TGTAGAATACTCCAGCAACGTT |
| GluR3 R | ACGTGGTAGTTCAAATGGAAGG |
| GluR3 F | ACCATCAGCATAGGTGGACTT |
| mNCOA3 F | AGTGGACTAGGCGAAAGCTCT |
| mNCOA3 R | GTTGTCGATGTCGCTGAGATTT |
| mNCOA1 F | CATCAGACCCTGCAAACCCA |
| mNCOA1 R | GTTGGCAGACAGTAGTTCAG |
| mNCOA2 F | GCCCTCGATGGGTTCTTCTTC |
| mNCOA2 R | CCTGGGAGGTTCTCCAGAC |

**Table S2.** The sequences of primers for construction different truncated Tet2 promoters

|  | Sequence (5’→3’) |
| --- | --- |
| Tet2pro-3663F | ggtaccgagctcttacgcgtCTTCTTACCCGATGTGCCAT |
| Tet2pro-1608F | ggtaccgagctcttacgcgtTCAGACATCCTACTGCCTCA |
| Tet2pro-XhoI R | cttagatcgcagatCTCGAGCTGGTTCTGGTCCCTCAGCC |

**Table S3**. The primer sequences for construction expression plasmid

| Gene Name | Sequence (5’→3’) |
| --- | --- |
| NR1D1 F | GTACCGAGCTCGGATCCATGACGACCCTGGACTCCAAT |
| NR1D1R | GAGATGAGTTTTTGTTCCTGGGCGTCCACCCGGAAGG |
| NRL F | ACCGAGCTCGGATCCATGGCTTTCCCTCCCAGTCCCTTGG |
| NRL R | GATGAGTTTTTGTTCGAGGAAGAGGTGTGTGTGGTCG |
| DHX30 F | TACCGAGCTCGGATCCATGGTGACTCCTGTCTGTAATTC |
| DHX30 R | AGATGAGTTTTTGTTCGTCATCAGCTGTCTTGCGCA |
| CRXF | TACCGAGCTCGGATCCATGGCTCAGTGGTTAAGAACACT |
| CRX R | GAGATGAGTTTTTGTTCCAAGATCTGAAACTTCCAGGCA |
| LSD1F | TTGGTACCGAGCTCGGATCCATGTTGTCTGGGAAGAAGGC |
| LSD1R | ATCCTCTTCTGAGATGAGTTTTTGTTCCATACTTGGGGACTGCT |
| Sin3a F | GTACCGAGCTCGGATCCATGAAGCGACGTTTGGATGAC |
| Sin3a R | TCCTCTTCTGAGATGAGTTTTTGTTCAGGGGCTTTGAATACTGTGC |
| mNCOA1 F | TTGGTACCGAGCTCGGATCCATGAGTGGCCTTGGGGACAG |
| mNCOA1 R | GAGATGAGTTTTTGTTCGAATTCAGTCAGTAGCTGCTGAAG |
| mNCOA2 F | TTGGTACCGAGCTCGGATCCATGAGTGGGATGGGAGAAAAC |
| mNCOA2 R | CTGAGATGAGTTTTTGTTCGAAGCTTATTCTAGAGTACGAAG |
| mNCOA3 F | TTGGTACCGAGCTCGGATCCATGAGTGGACTAGGCGAAAG |
| mNCOA3 R | CTGAGATGAGTTTTTGTTCGAAGCAGTATTTCTGATCGGGGC |

**Table S4.** The primers of Tet2 promoter

|  | Sequence (5’->3’) |
| --- | --- |
| Tet2-11.3F | CCAAACCAAACCTGCAGTAC |
| Tet2-11.3R | AGGAGGGAAACGTGAGTAGC |
| Tet2-10.3F | CAGTTCTCTCATTCCATCTC |
| Tet2-10.3R | GATACTATGACCCACATGTG |
